# Human endothelial cells display a rapid tensional stress increase in response to tumor necrosis factor-*α*

**DOI:** 10.1371/journal.pone.0270197

**Published:** 2022-06-24

**Authors:** Matthias Brandt, Volker Gerke, Timo Betz

**Affiliations:** 1 Institute of Cell Biology, Center for Molecular Biology of Inflammation, University Münster, Münster, Germany; 2 Institute of Medical Biochemistry, Center for Molecular Biology of Inflammation, University Münster, Münster, Germany; 3 Third Institute of Physics—Biophysics, Georg August University Göttingen, Göttingen, Germany; Normandie Universite, UNITED STATES

## Abstract

Endothelial cells form the inner layer of blood vessels, making them the first barrier between the blood and interstitial tissues; thus endothelial cells play a crucial role in inflammation. In the inflammatory response, one important element is the pro-inflammatory cytokine tumor necrosis factor-*α* (TNF-*α*). While other pro-inflammatory agents like thrombin and histamine induce acute but transient changes in endothelial cells, which have been well studied biologically as well as mechanically, TNF-*α* is primarily known for its sustained effects on permeability and leukocyte recruitment. These functions are associated with transcriptional changes that take place on the timescale of hours and days. Here, we investigated the early mechanical action of TNF-*α* and show that even just 4 min after TNF-*α* was added onto human umbilical vein endothelial cell monolayers, there was a striking rise in mechanical substrate traction force and internal monolayer tension. These traction forces act primarily at the boundary of the monolayer, as was to be expected. This increased internal monolayer tension may, in addition to TNF-*α*’s other well-studied biochemical responses, provide a mechanical signal for the cells to prepare to recruit leukocytes.

## Introduction

Endothelial cells (ECs) constitute the interior surface of blood vessels and form the key barrier between the blood and the surrounding tissues, ensuring, among other things, that blood cells do not inadvertently enter. In this function, ECs are known to regulate tissues’ exchange of molecules and fluid, but they also regulate the entrance of immune cells and, in pathological situations, even metastatic cancer cells. Hence, this gatekeeper function plays an important role in physiological maintenance not only of the vascular system, but of all the tissues in general.

ECs are constantly challenged by mechanical stress due to blood pressure, changes in surrounding tissue stiffness and vessel stretch. Therefore, over the last few decades, researchers have thoroughly studied the importance of mechanical stress in vascular diseases [1, 2]. While many inflammatory processes are known to increase EC monolayer’s mechanical tension and barrier permeability [2, 3], the mechanical force load on the ECs might, in itself, alter the immune response and affect the proper guidance and migration of immune cells into injured tissues [4, 5]. However, in stark contrast to the well-described importance of mechanical forces and endothelial mechanics for proper immune responses, we have only sparse knowledge about the endothelial cells’ early mechanical responses to inflammatory signals, such as tumor necrosis factor-*α* (TNF-*α*). Although ECs are known to have a rapid chemical reaction to TNF-*α*, the link to a mechanical reaction has only been established over relatively long time frames.

TNF-*α* is one of the central pro-inflammatory signaling molecules and is mostly supplied by macrophages and monocytes that have been activated via pathogen interaction. A prominent function of TNF-*α* is to increase endothelial permeability and to sustain enhanced leukocyte recruitment [6–8]. While these effects are mostly associated with transcriptional changes, thus taking place on a slow timescale of several hours, other pro-inflammatory agents like thrombin and histamine induce acute transient changes in ECs that take place within minutes [9, 10]. For example, biophysical studies measuring either substrate traction forces exerted by ECs on elastic hydrogels or monolayer tension using isometric force transducers confirmed that thrombin quickly and strongly enhances EC monolayer contractility which is associated with increased cell-cell forces [11, 12]. On the other hand, for TNF-*α*, biophysical studies have focused mostly on long-term changes (some in only single cells instead of interconnected monolayers) within 4–24 h after exposure, where changes have involved reduced cortical stiffness, cell elongation and enhanced cell-substrate forces [13–16].

Yet, some studies on TNF-*α* have shown that rapid morphological changes occur, similar to but weaker than the response to thrombin. TNF-*α* was found to induce actin cytoskeletal reorganizations within the first 5–10 min of stimulation, i.e., enrichment of fine actin cables and the formation of lamellipodia and filopodia [17]. After 15–30 min, these changes were followed by the increased formation of stress fibers, and, at the same time, ECs began to retract and VE-cadherin started to dissociate from intercellular junctions. The formation of stress fibers in response to TNF-*α* has been linked to increased levels of RhoA activity promoting myosin light chain (MLC) phosphorylation via downstream activation of Rho-associated kinase (ROCK) [18, 19]. In turn, MLC phosphorylation is known to increase actomyosin contractility [20].

However, the mechanical response in form of force generation onto the substrate and on cell neighbors remains to be explored. Recently, the mechanical interaction not only with the substrate has been shown to be important for cell layers’ mechanical equilibrium, but also the force transmission to the neighboring cells via cell-cell adhesion has become increasingly investigated to better understand the biomechanics of cell layers in general [21–23] and endothelial cells in particular [11, 24–27]. To directly investigate the mechanical interactions between ECs, their substrates and their neighbors, we used patterned monolayers combined with traction force microscopy to gain access to both the substrate traction and the cell-cell forces exerted by endothelial cells.

## Results and discussion

### Circular HUVEC monolayers apply substrate stress primarily at their boundary

**Fig 1:**
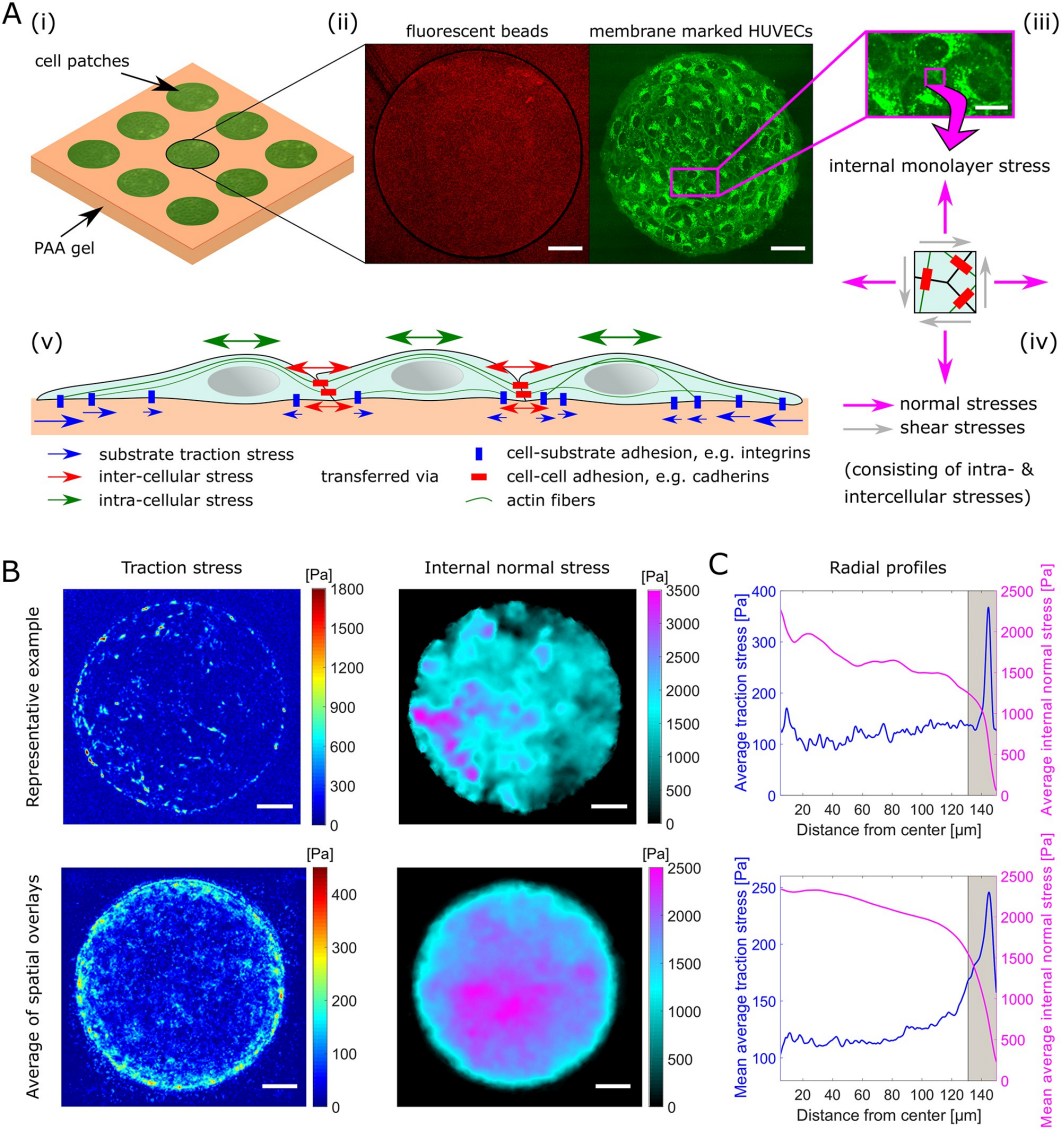
Experimental system and mechanical stress distributions. **A**: Circular islands of EC monolayers reside on (**i**) micropatterned PAA gels that (**ii**) incorporate fluorescent beads (red) close to the surface for traction force microscopy. (**iii**) Zoom in on plasma membrane stained (CellMask Deep Red) HUVECs in a monolayer patch (in **ii**—green). (**iv**) Normal and shear stresses act at any position within a monolayer of cells. (**v**) Schematic illustration of mechanical stress exerted and transmitted by interconnected cells. Scale bars, 50 μm (15 μm for zoomed-in image). **B**: Color-coded maps of derived traction stress and internal normal stress for a representative example monolayer patch (top) and for the spatially overlayed and averaged total of 22 different patches from 3 independent experiments (bottom). Since the spatial averaging reduces the signal-to-noise ratio in the overlayed images, a force level threshold was applied to those images prior to the averaging for better visualization. Scale bars, 50 μm. **C**: Corresponding radial profiles of average traction and internal normal stress based on the data used in B (top for single example, bottom for average of multiple patches). The gray marked area indicates the approximate region of edge cells. The inner 5 μm have been neglected here due to poor number of pixels statistics. Traction stresses are predominantly applied by cells at the edge of the monolayers, while internal normal stresses accumulate toward the center.

To determine the mechanical response of a human umbilical vein endothelial cell (HUVEC) monolayer upon exposure to TNF-*α*, traction force microscopy was performed on cells seeded overnight on a polyacrylamide (PAA) gel of 15 kPa stiffness and grown in circular patches. The circular-shaped monolayers were formed via micropatterning of a collagen coating on the gel surface; monolayers had a diameter of 300 μm and were distributed over a periodic grid with 150 μm edge-to-edge spacing between the circular cellular islands (Fig 1A). This pattern size was chosen to allow for the formation of bigger cell clusters resembling a monolayer, while maintaining a small enough pattern to fit into the microscope’s field of view when using a 40X water immersion objective. For initial experiments on stained cells shown in Fig 1, images were instead recorded with a 60X objective with higher numerical aperture and stitched together for better quality. The size was optimized for rapid image acquisition of several patterns during a single experiment, and the distance between patterns was sufficient to exclude measurable interactions between neighboring patterns (see S1 File). Although the pattern is small, it should exhibit approximate monolayer properties based on [28].

The pattern allowed us to differentiate between the mechanical forces acting on a substrate in the region of the intact monolayer, and those forces acting at the edge regions of the pattern, where the monolayer was disrupted and an open edge exists. Consistent with reports on epithelial cells [29, 30], we found the traction forces to be focused at the monolayer edge (Fig 1B). To quantify this, we compared average forces exerted by cells at the edge and those exerted by cells in the inner region of the pattern. The difference between the edge and inner cells was defined as the manually measured average cell diameter of the edge cells, which was about 19 μm. Using this radial distance from the pattern edge, we found that the edge cells exerted average traction stresses of ≈198 Pa, which was 65% higher than those exerted by the inner cells (≈120 Pa; Fig 1C). Notably, the background level was at about 100 Pa, making this discrepancy even more striking.

The reason for such a behavior, in general, can be explained by one of two hypotheses. First, it is possible that only the cells at the periphery are contractile, such that only these apply a reasonable force on the substrate. Alternatively, in a monolayer forces can also be passed from cell to cell by intercellular junctions, in particular adherence junctions. Since cells at the edge lack neighboring cells to pass forces onto, they would be required to transmit the eventual forces they received from their inner neighbors onto the substrate, in order to achieve force balance. This second explanation has previously been demonstrated to be correct in other monolayer systems [29] and, hence, was tested for the present system.

### Transcellular stress transmission in the monolayer region dominates traction forces

To assess the stresses passed from cell to cell we employed monolayer stress analysis similar to that introduced previously [21, 24]. In short, internal monolayer stresses are inferred from measured traction forces by imposing a force balance under the assumption of homogeneous material properties throughout the whole cell monolayer. As expected, and consistent with previous reports on other monolayer systems, we found high internal monolayer stress transmission throughout the whole circular pattern, with high stress accumulation at the central region of the pattern (Fig 1B). This accumulation might be a geometrical effect that arises from the shape of the pattern and does not necessarily reflect the real stress distribution throughout a general HUVEC monolayer. The internal stress maps shown here give the average of only the normal component, which in general, represents either tensional (positive values) or compressive (negative values) stresses as opposed to shear stresses, which run parallel to a surface (see Fig 1A-(iv)).

When again comparing the average values of the edge vs. the inner cells, now for the tensional monolayer stress, we see the opposite of what was found for the traction forces. For the tensional stress, the inner cells have two times larger average stresses, with ≈2.1 kPa, than the cells at the edge of the pattern, which have average stresses of ≈1.0 kPa.

Our observation that the central region of the monolayer shows low substrate traction forces but high internal monolayer stresses, whereas the cells at the edge show the opposite (high traction forces but low tensional stresses), is best explained by a high transcellular stress transmission through the monolayer. Further, because the difference between the outer and inner cells is striking at just one cell diameter of 19 μm away and plateaus at about 50 μm away from the edge of the pattern, this may mean that the endothelial cells prefer to transmit stresses among themselves instead of applying forces on the substrate; yet, the current approach is unable to determine the precise force transmission that is passed from cell to cell via stress fibers. Finally, one interesting speculation for why force transmission is eventually shifted from cell-cell contacts to substrate contacts might be via the generation of defects or holes in a monolayer, for example during high stress within blood vessels or because of toxins. Such changes in force transmission, which are purely mechanical effects and independent of molecular signaling, would effectively allow a cell to recognize monolayer rupture by the simple engagement of substrate forces.

### TNF-*α* increases traction forces and transcellular stress

Having established a method to determine both the traction forces and the internal monolayer stresses of an endothelial cell layer, we went on to investigate potential changes of the mechanical properties of HUVEC monolayers in response to TNF-*α* signaling. It is well established that long-term application of TNF-*α* leads to prominent stress fiber formation and increased permeability in EC monolayers. Investigations on single cells have also found that TNF-*α* induces a long-term increase in contractility mediated by substrate forces [14]. However, the extent to which the overall stress distribution inside a monolayer is affected by TNF-*α* had not been studied yet.

**Fig 2:**
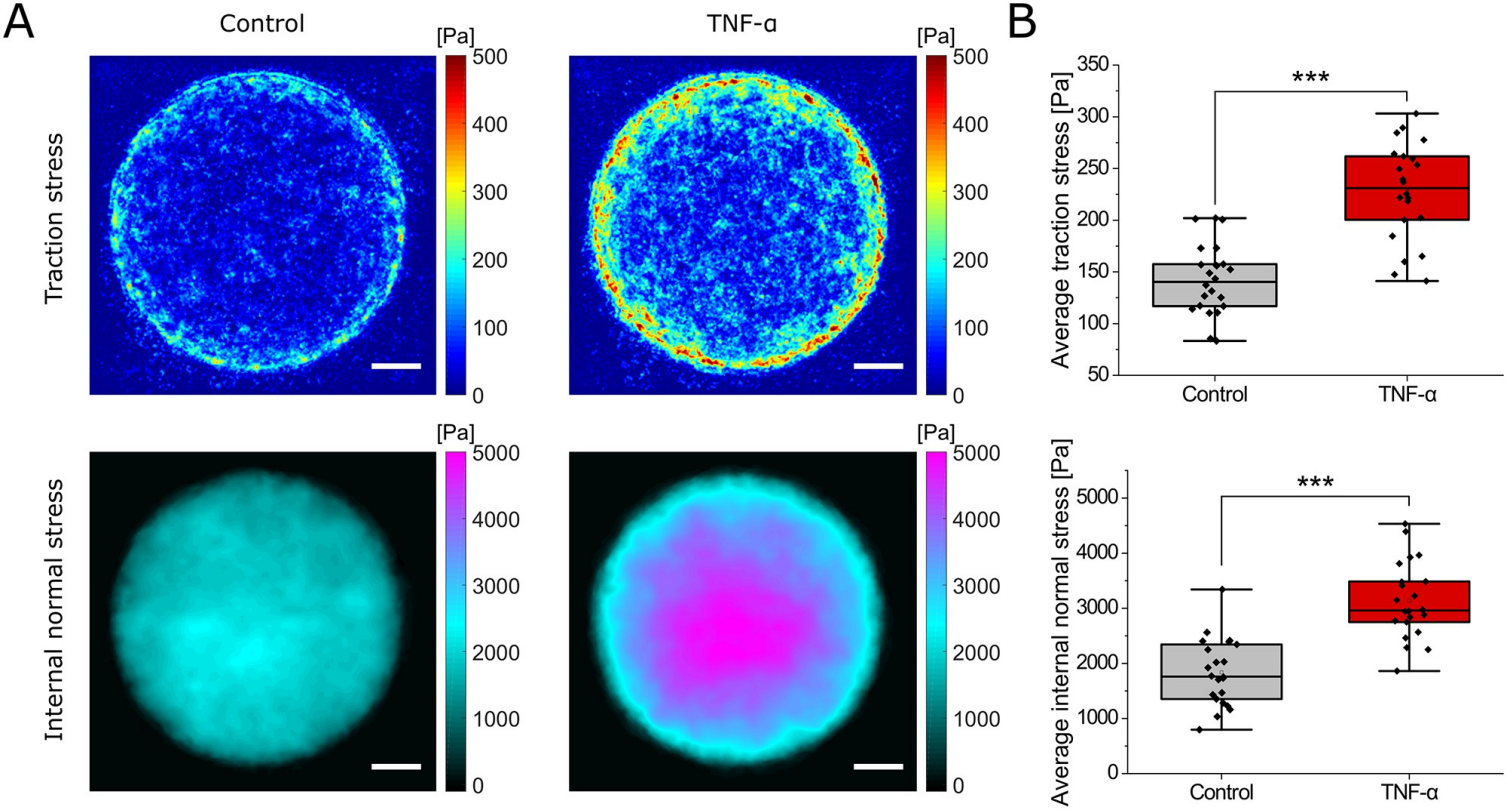
Increase of both traction and internal normal stress upon exposing HUVECs to 20 ng/ml TNF-*α* for 1 h. **A**: Spatially overlayed and averaged color-coded maps of derived substrate traction and internal normal stress of multiple monolayer patches prior to (Control) and upon application of TNF-*α*. Since the spatial averaging reduces the signal-to-noise ratio, a force level threshold was applied prior to the averaging for better visualization. Scale bars, 50 μm. **B**: Average traction stress (top) as well as internal normal stress (bottom) increased significantly after 1 h of TNF-*α* treatment. Data for A and B is based on *n* = 22 monolayer islands from *N* = 3 independent experiments; ***, *p* < 0.001.

As presented in Fig 2B, after applying TNF-*α* for 1 h at a 20 ng/ml concentration, we found a significant increase of both, the average substrate traction forces as well as the average internal normal stress throughout the monolayer. Both, the traction forces and the internal stresses increased similarly in magnitude, and no qualitative change in the force distribution was observed. Similar to control conditions, the traction forces occurred predominantly at the edge, while normal stresses within the layer built up toward the center (Fig 2A). Together, these findings hint that the whole cell monolayer patch contracts through a joint effort. This is further supported visually by comparing the fluorescent images of membrane stained cells in both conditions (S1 Video).

### Stress increase upon TNF-*α* stimulation occurs on a rapid timescale

**Fig 3:**
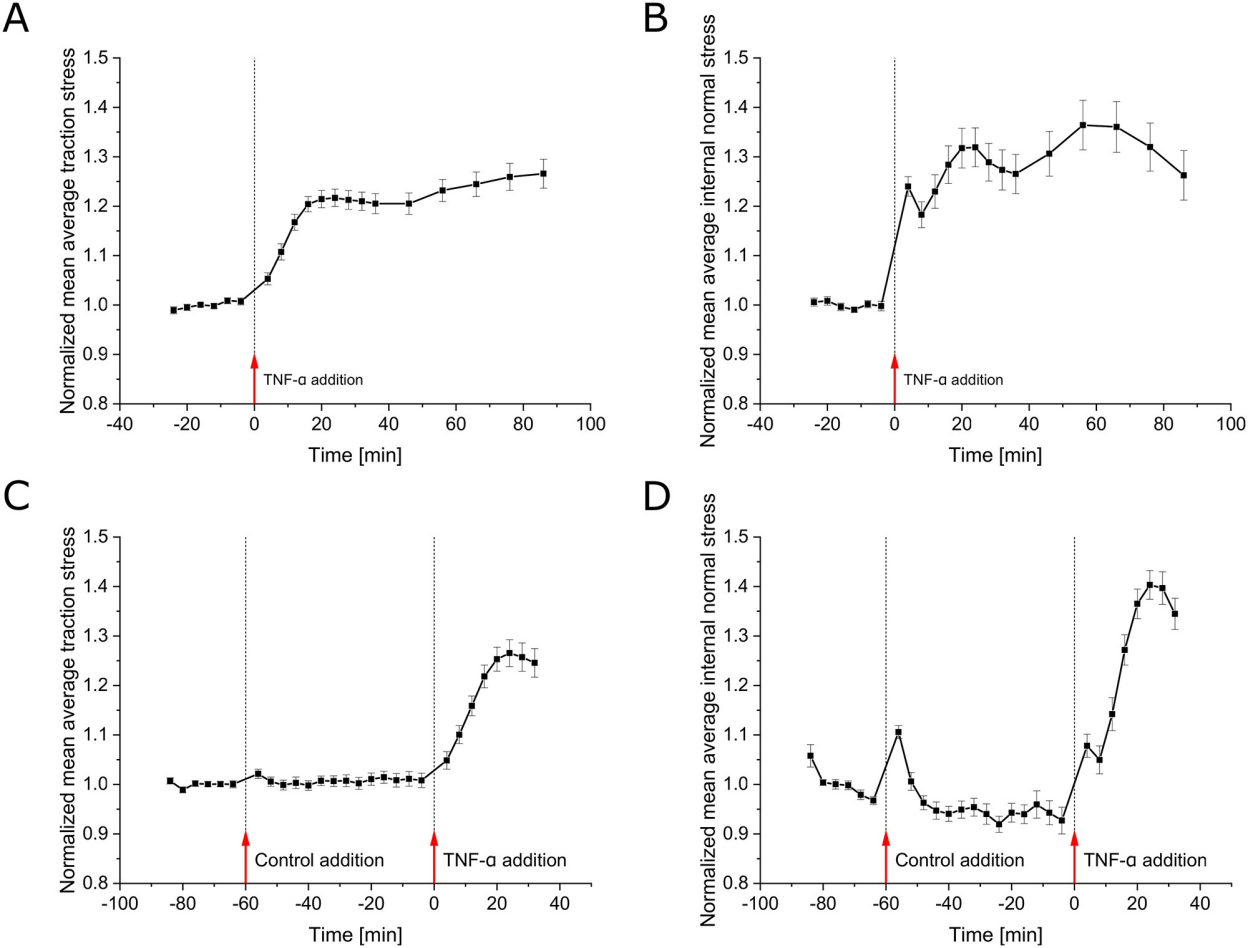
Time evolution of the mechanical response of HUVECs upon TNF-*α* exposure. **A,B**: Time course of the mean of spatially averaged (A) traction stress and (B) internal normal stress of multiple monolayer islands recorded at baseline and upon 20 ng/ml TNF-*α* treatment. Data is shown as mean ± SEM and has been normalized to the mean of the first 6 time points recorded prior to cytokine administration for each individual monolayer island (*n* = 29, *N* = 3). Average stresses rise immediately upon TNF-*α* treatment (*p* = 0.0021 for traction stress at 4 min vs. -4 min), reaching a peak after about 20 min. While substrate traction stress increases monotonically, for the internal normal stress a quick steep rise (*p* < 0.001 at 4 min vs. -4 min) with a subsequent intermediate drop (*p* = 0.027 from 4 to 8 min) can be observed. The peak after 20 min reaches slightly higher for internal normal stresses than for traction stresses (31.8% vs. 21.4% relative increase). **C,D**: Same as A,B, respectively, but for control experiment. After again recording a 20 min baseline, as control only the solvent (water) without TNF-*α* was applied. To the same samples TNF-*α* was added 1 h later at *t* = 0. Data is shown again as mean ± SEM and has been normalized to the mean of the first 6 time points for each individual monolayer island (*n* = 27, *N* = 3). While the control addition had only a barely visible effect on the average traction stress, the average internal normal stress increased significantly after one time frame (*p* < 0.0001 at -56 min vs. -64 min). This increase was followed by a quick decay reaching prior levels after three time frames. The later addition of TNF-*α* to the same samples caused, qualitatively, the same response as in the previous experiments without prior control administration while reaching slightly higher levels. Only the initial peak in the internal normal stress was smaller but was of about the same magnitude as in the case of the control addition.

To better understand the time scale of the mechanical response we recorded the dynamics of contractility by taking full 3D stacks of cells and substrate deformation for multiple patches with a 4 min time resolution. To avoid photodamage on the cells due to the prolonged exposure of fluorescent membrane markers, cells were only observed using bright-field illumination. In these experiments, we initially recorded the cells and the deformation for 20 min to obtain a force baseline. Subsequently, we added 20 ng/ml TNF-*α*. As shown in Fig 3A and 3B, we found an immediate response, as the average stresses started to rise even within the first acquired time interval (*p* = 0.0021, *p* < 0.0001 at -4 min vs. 4 min for traction and internal normal stress, respectively), with a typical peak at about 20 min after applying the cytokine. The magnitude of this peak was slightly higher for the internal normal stress than for the traction stress (31.8% vs. 21.4% relative increase), suggesting an enhancement in transcellular stress transmission. As the absolute stress levels varied substantially between individual cellular islands, we chose here and in the following analyses to normalize all stresses by their baseline, to prevent any individual island from dominating the outcome.

Further, while the average traction stress increased monotonically, we were surprised to observe that the average internal monolayer stress exhibited a short-lived stress peak immediately after the cytokine was added; this peak relaxed significantly (*p* = 0.027) at the next measured point (Fig 3B). As our administration of TNF-*α* might have induced shear stress onto the cells through fluid flow due to the involved aspiration and restitution of two-thirds of the medium, we performed a control experiment by applying the same amount of solvent (0.6 μl of water to 3 ml medium) without the TNF-*α*. While the average traction stress showed only a barely observable slight increase, the average internal normal stress showed a significant (*p* < 0.0001) increase of about 10% after one time interval of 4 min (Fig 3C and 3D). The increase in the average internal normal stress immediately started to decay, and stresses reached prior levels after 12 min. To ensure that the TNF-*α* effect was still observed in these controls, we administered TNF-*α* after 1 h to the same samples, again at a 20 ng/ml concentration. Consistent with the previous measurements (Fig 3A and 3B), we again found an increase in both the average traction stress and internal normal stress, that also matched the previously observed temporal development. The initial peak in the average internal normal stress showed a similar amplitude as in the control addition. These findings strongly suggest that the acute intermediate peak in internal stress after the first time interval was a result of the administration process, while the ongoing increase can indeed be attributed to TNF-*α* signaling.

Next, we wondered whether the rapid changes in mechanical stress might be visibly reflected by changes in the actin cytoskeleton. Therefore, we acquired immunofluorescence images marking actin filaments with a phalloidin stain for the first 20 min of exposure at time intervals of 4 min (see S1 Fig). However, due to substantial variability in the individual patterns and the lack of a quantitative measure, we were not able to identify a systematic temporal development in actin cytoskeletal structure.

### Acute transcellular stress redistribution is avoided in the case of constant hydrodynamic stress

**Fig 4:**
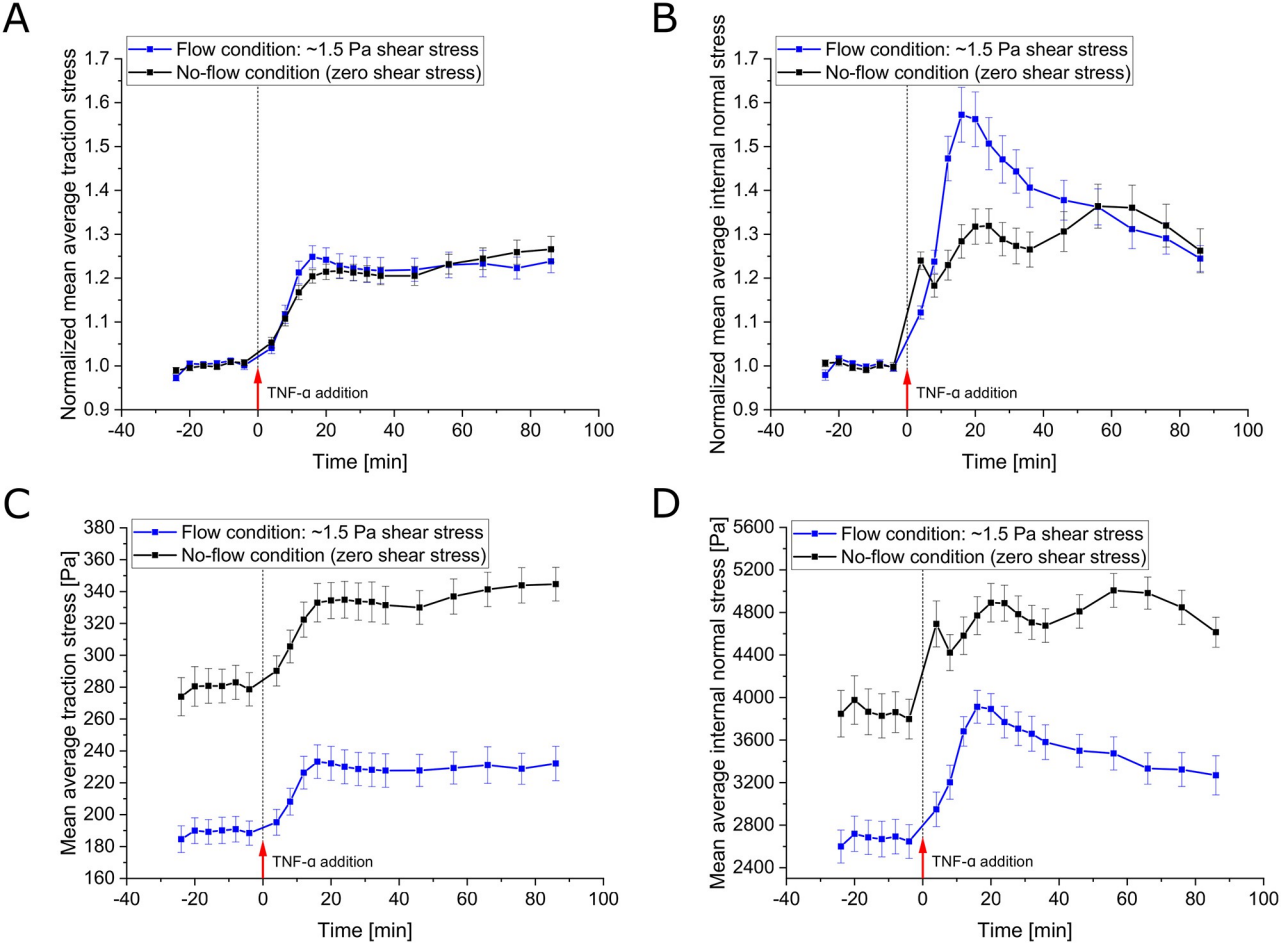
HUVECs’ mechanical response upon TNF-*α* exposure in flow (1.5 Pa shear stress) and no-flow (zero shear stress) conditions. **A,B**: Time course of the mean of spatially averaged (A) traction stress and (B) internal normal stress of multiple monolayer islands recorded at baseline and upon 20 ng/ml TNF-*α* treatment. Data is shown as mean ± SEM and has been normalized to the mean of the first 6 time points recorded prior to cytokine administration for each individual monolayer island (*n* = 35, *N* = 3 under the condition of flow-induced shear stress; for the no-flow condition data are the same as in Fig 3A and 3B). The time course of the average traction stress in response to TNF-*α* under flow conditions closely follows the one observed in the case of no flow, with the exception of a slight overshoot of peak values. The initial steep rise with a subsequent intermediate drop in internal normal stress is, as expected, absent in the presence of constant fluid flow. Interestingly, the level of the actual peak reached in response to TNF-*α* is significantly higher than in the situation without applied flow (*p* = 0.0022 at 20 min peak). **C,D**: Same as in A,B, respectively, but the averages of absolute stress values are shown instead of normalized data. The absolute stress values are substantially lower in the presence of fluid flow-induced shear stress. Even after the higher relative increase in internal normal stress in response to TNF-*α*, values do not exceed those observed for the no-flow condition; in fact, the maximum levels only reach the baseline levels observed in the no-flow condition.

As the procedure of administering TNF-*α* caused the cells to temporarily change their internal stress distribution—supposedly by inflicting shear stress by fluid flow—we wondered whether the initial peak in internal monolayer stress could be effectively avoided by establishing a continuous fluid flow prior to adding TNF-*α*. HUVECs are exposed to fluid flow in their natural environment, and previous studies focusing on fluid shear stress reported that ECs align in the direction of flow and undergo remodeling of their cytoskeleton and intercellular junctions via mechanotransduction pathways [31–36]. Therefore, it is an interesting question whether ECs’ mechanical response to TNF-*α* might be altered under flow conditions. To this end, before the measurements were conducted, HUVECs were seeded on PAA gels residing at the bottom of a flow chamber, allowed to spread and grow and were then exposed for about 16 h to high rates of laminar flow that induced about 1.5 Pa (15 dyn/cm^2^) shear stress onto the cells. The flow rate was kept this high throughout the measurement (apart from short interruptions during mounting and addition of TNF-*α*). Qualitatively, the traction stresses responded similarly to TNF-*α* in the flow and no-flow conditions (Fig 4A). However, in the presence of constant fluid flow-induced shear stress, the initial rapid peak found in the internal monolayer stress was absent (Fig 4B). This further supports the idea that our prior administration system did indeed introduce turbulence, albeit short and relatively small, to the surrounding medium that acutely affected stress transmission within the monolayer.

### Higher relative increase of internal monolayer stress upon TNF-*α* stimulation but lower base level in the presence of fluid shear stress

Interestingly, while the amplitude of the average traction stress peak was comparable with and without fluid flow, we found a significantly higher internal normal stress peak in the presence of flow (Fig 4B, ∼56% vs. 32% relative increase at *t* = 20min, *p* = 0.0022). In previous studies, intercellular stresses have been found to start aligning with the direction of fluid flow after 1 h of fluid flow application, and subsequently they slowly attenuated in amplitude over time [26]. This could explain the higher relative increase in response to TNF-*α* observed here: on the one hand, because a higher degree of stress alignment might enhance the efficiency of stress transmission, and on the other hand, because a lower base level of stress might allow for a stronger increase in intercellular stress without exceeding the maximal force that can be transmitted between cells before causing rupture in cell-cell adhesion. Comparing the absolute traction and internal stress values in both situations, we found that indeed the level of traction stress and internal monolayer stress was substantially lower in the presence of flow (Fig 4C and 4D). Even after the higher relative increase in internal normal stress in the case of fluid shear, the absolute magnitudes still remained below those observed in the case of no flow and, hence, zero shear stress.

### Delayed increase of central traction forces is reduced by hydrodynamic pre-stress

**Fig 5:**
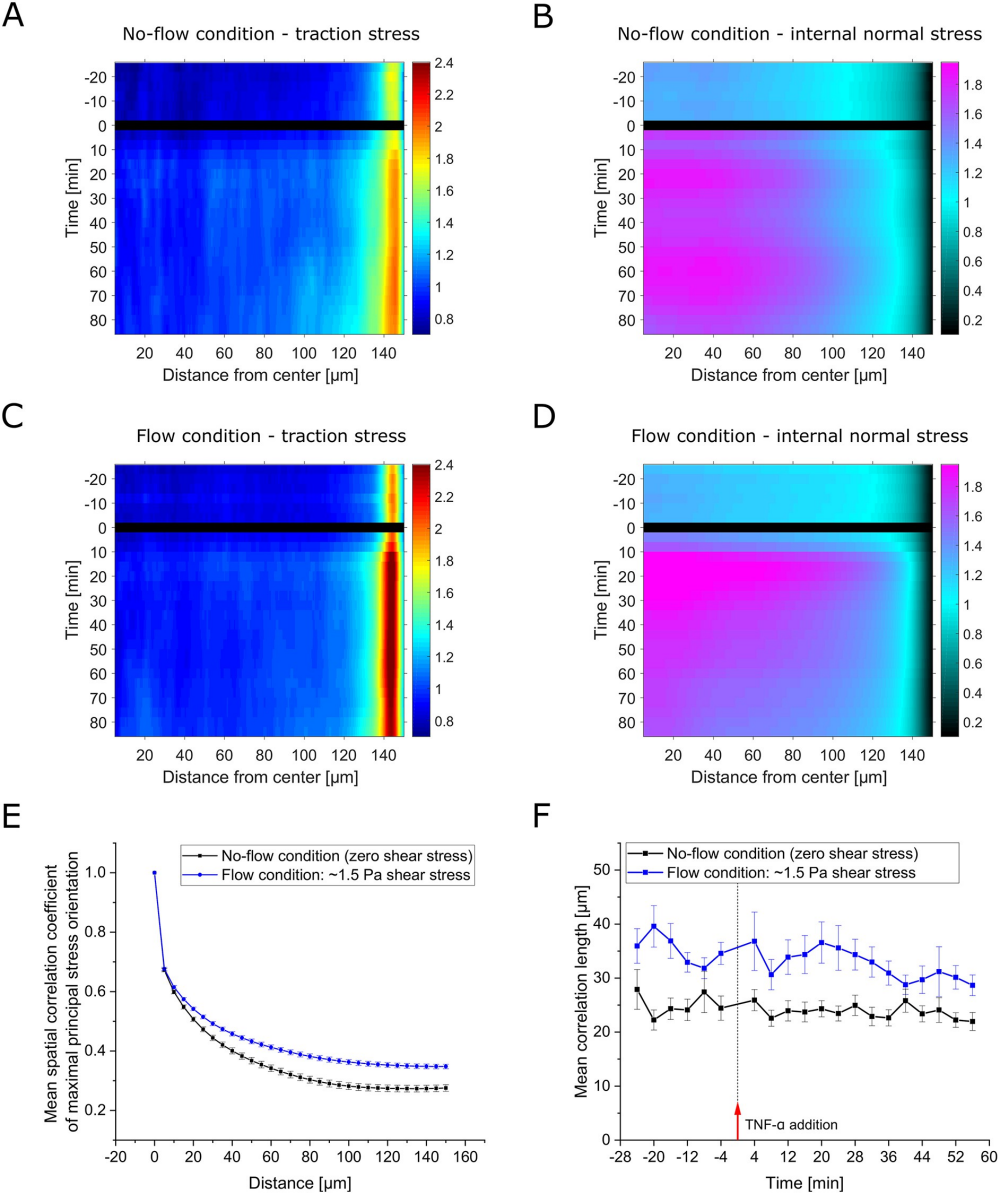
Spatial distribution and cooperativity of stress in response to TNF-*α* exposure under flow (1.5 Pa shear stress) and no-flow (zero shear stress) conditions. **A-D**: Kymographs showing the development of radial profiles of (A,C) substrate traction and (B,D) internal normal stress over time under (A,B) no-flow (zero shear stress) conditions and (C,D) flow (1.5 Pa shear stress) conditions. Kymographs are based on the same measurements as data in Fig 4A and 4B. Colors encode the stress intensity normalized as in Fig 4A and 4B. As application of TNF-*α* required some time and caused disturbance, no image data was analyzed for the respective timepoint 0 (black line). The overall increase in stress intensity fits the time course of the average values shown in Fig 4A and 4B. While the rapid increase in traction stress is largely generated at the edge of the monolayer, a delayed increase of traction stress in the inner region can still be observed (see A,C). Interestingly, the discrepancy between edge and inner region of the monolayer for the exerted traction stress is significantly higher in the presence of flow (see also S2 Fig). The initial rise inflicted by the administration process is apparent throughout the inner region of the monolayer (see B). In agreement with the average values, this is fully absent in (D) the situation of unaltered external stress in the presence of fluid flow. The higher relative increase in the condition of fluid shear stress is observable throughout the whole inner region. However, the subsequent decay in this case, appears even more striking than expected from the average values. Conversely, in the situation of no flow, the internal normal stress in the inner region starts to oscillate. **E**: Mean spatial correlation coefficient of maximal principal normal stress orientation under flow (1.5 Pa shear stress) and no-flow (zero shear stress) conditions as a function of the distance between stress locations. The coefficient was calculated for and averaged over all time points and individual cellular islands from all 3 experiments, respectively for both conditions. The error shown is the SEM with respect to individual islands. The level of correlation of stress orientations is higher in the presence of fluid flow. **F**: Mean correlation length for individual time points in both conditions. The correlation length has been defined as the distance at a value of one half of the correlation coefficient of maximal principal normal stress orientations. No clear trend can be identified.

To better understand how the spatial distribution of both the traction stress and the internal monolayer stress develop, we generated kymographs of the radial stress profiles (Fig 5A–5D). In these kymographs, each horizontal line represents the radial stress profile at the given time, and the color encodes for the normalized stress intensity. As the addition of TNF-*α* at time 0 briefly disturbed the measurement, the corresponding images cannot be analyzed (black line). As expected, the radial kymographs show an increase in both traction stress and internal monolayer stress after TNF-*α* addition, which is consistent with the overall time evolution of the stresses. The kymographs show that the described rapid increase in traction stress is largely generated at the edge of the monolayer for both the situation with and without flow-induced shear stress (Fig 5A and 5C). Surprisingly, a closer inspection of the traction force kymographs for both conditions shows differences in the inner region of the monolayer patch. While in the situation with applied flow and, hence, shear stress, only a marginal traction stress increase in the monolayer region is found, this increase is much more pronounced in the situation without shear stress. This difference becomes even more striking when comparing the relative changes of traction forces of edge and inner region in the two conditions (see S2 Fig).

### Quicker, relatively stronger transcellular stress alterations and higher stress cooperativity in the presence of fluid shear stress

Turning to the kymographs of the internal monolayer normal stress (Fig 5B and 5D), we confirmed the initial rise due to the administration process in the situation without fluid flow (*t* = 4min, Fig 5B). This rapid increase acts throughout the inner region of the monolayer and is absent in the case of fluid flow (Fig 5D). In the case of fluid flow-induced shear stress, we noticed a stronger relative increase but also a more rapid decay of stresses in the inner region of the monolayer. This change in the inner region appears even more striking than for the average values. While in the case of fluid flow the stresses continuously decay after reaching a peak, in the case of zero external shear stress they start to oscillate. Together with the large difference in traction stresses between the edge and inner region, the striking relative changes in internal stress throughout the monolayer suggest, that in the situation of fluid flow, transcellular stress transmission is particularly strongly redistributed. This quick redistribution might be supported by an alignment of stresses with the direction of flow, which was previously reported [26], which might naturally also increase the alignment of stresses between neighboring cells. To investigate this, we analyzed the distance-dependent correlation of maximal principle stress orientations within the monolayer. This measure quantifies the extent to which cells at a certain distance show similar orientations of internal stress. A high level of cooperativity between cells within the monolayer would imply that even at longer distances cells show similar behavior, which is typically quantified by a correlation length. The average distance-dependent correlation was indeed higher in the fluid shear stress condition as compared to the no-flow condition (Fig 5E). Analyzing the correlation length for different time points, we did not observe significant (according to the Kruskal–Wallis test, *p* = 0.996 for no flow and *p* = 0.277 for the flow condition) changes over time in either condition, not even after TNF-*α* treatment (Fig 5F). The difference between both conditions, however, proves to be highly significant (*p* < 0.0001) with a higher average correlation length in the presence of fluid shear stress of 33.3 μm, compared to 24.1 μm in the case of no fluid flow. This suggests an increased cell-cell cooperativity under flow conditions, which might be mediated by mechanical forces transmitted between cells.

### Qualitatively similar but delayed response of HUVEC monolayers with decreasing TNF-*α* concentration

**Fig 6:**
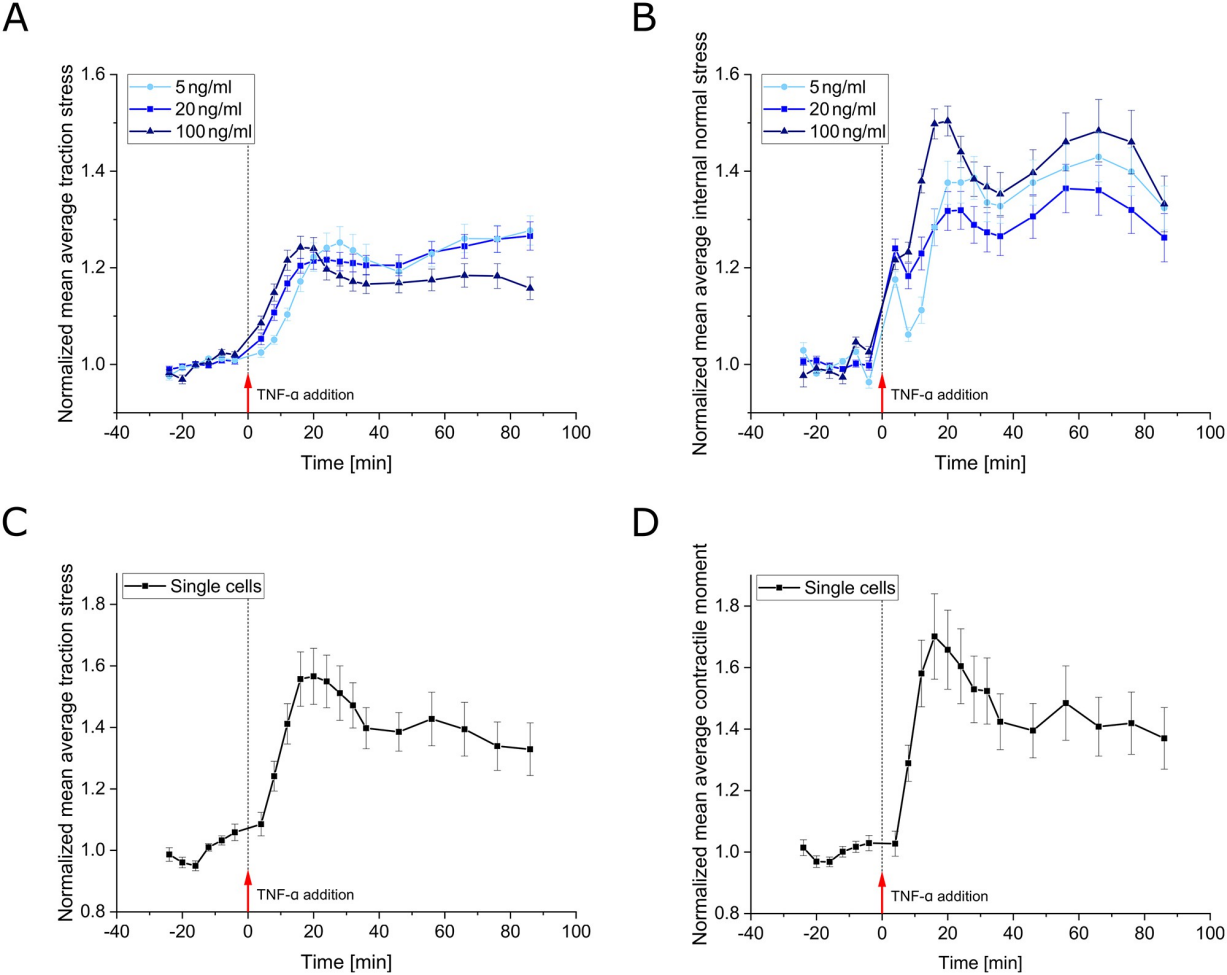
Time evolution of the mechanical response upon TNF-*α* exposure for different cytokine concentrations and for single cells. **A,B**: Time course of the mean of spatially averaged (A) traction stress and (B) internal normal stress of multiple monolayer islands recorded at baseline and upon TNF-*α* treatment for 3 different concentrations as indicated. Data is shown as mean ± SEM and has been normalized to the mean of the first 6 time points recorded prior to cytokine administration for each individual monolayer island. For each concentration, 3 independent experiments were performed with a respective number *n*_5ng_ = 29, *n*_20ng_ = 29 or *n*_100ng_ = 33 of monolayer patches. No clear difference in strength of the response can be observed for the different concentrations. However, the response appears to occur slightly faster with increasing cytokine concentration. As a result, the drop in internal normal stress after the first steep rise, which is caused by our administration procedure, appears progressively reduced. **C**: Same as A, but for single endothelial cells instead of EC monolayers and at a 20 ng/ml TNF-*α* concentration only (*n* = 26, *N* = 3). Single ECs display, qualitatively, the same response to TNF-*α* as EC monolayers while reaching significantly higher peak values (*p* < 0.001 at 20 min peak). **D**: Same as C, but the contractile moment is shown instead of the traction stress for the same cells as in C. Here, the contractile moment serves as a measure of intracellular stress as compared to the internal normal stress in the case of monolayers of ECs. However, no initial intermediate peak is found here but the time course rather matches the one observed for the average traction stress.

To test whether the ECs’ mechanical response to TNF-*α* stimulation might be dose dependent, we applied three different concentrations, namely 5, 20 and 100 ng/ml TNF-*α* to the HUVEC monolayers. As presented in Fig 6A and 6B (for unnormalized data, see S3A and S3B Fig), the qualitative response was independent of the concentration, though, interestingly, we did observe an overall delay of the response with decreasing concentrations. However, the magnitude of the response did not reveal a systematic trend. As expected from the earlier-described control experiment (see Fig 3D), the rapid intermediate peak in internal monolayer stress was of similar magnitude for all concentrations, but the subsequent drop decreased with increasing concentration, which highlights a delay in the response to TNF-*α*, thus making the peak inflicted by our administration procedure more pronounced for lower TNF-*α* concentrations.

### Single cell response is attenuated by contact in monolayers

Though introduced by the administration process, the qualitatively different initial behavior of the internal monolayer stress, led us to wonder whether and to what extent collective effects might be separated from the single cell response to TNF-*α*. To this end, we seeded single cells on the same elastic substrates and measured their relative increase in traction forces upon TNF-*α* stimulation at a 20 ng/ml concentration. As shown in Fig 6C, we found a qualitatively similar response of single ECs upon TNF-*α* stimulation, but the relative increase of stress was >50% larger (and significantly so, *p* < 0.001) than the increase of traction stress measured for the monolayers. However, this difference became marginal after an ongoing presence of TNF-*α*. These results suggest that in the monolayer situation, TNF-*α* stimulates an increase of force transmission to the neighboring cells, but this transmission might not ultimately change the average traction stress of the monolayer. A possible explanation for the observed difference could be found in intracellular mechanotransduction signaling in response to cell-cell force transmission, which can only occur within the monolayer. However, the details of such signaling effects need to be further studied. As a side note, when we consider the mechanical contractile moment of single cells as a measure of intracellular stress (Fig 6D), there was no initial internal stress peak occurring within single cells in contrast to the observed acute intermediate peak inside a monolayer on a transcellular scale. The unnormalized data is shown in S3C and S3D Fig.

### RhoA and Rac1 are both involved in TNF-*α* signaling that causes a rapid mechanical response

Since changes in protein expression require both transcriptional changes and translation of the newly generated mRNA, the observed rapid increase in mechanical stress is most likely explained by signaling that directly affects the present cytoskeletal structures. We therefore decided to perturb the intracellular signaling mediated by the small RhoGTPases RhoA and Rac1. While RhoA is generally known to control the activity of myosin motors and, therefore, to directly affect cellular contractility, Rac1 is known to be a key player in controlling the restructuring of the actin cytoskeleton. On top of that, RhoA and Rac1 have previously been reported to be involved in the formation of stress fibers and other organizational changes in the actin cytoskeleton in HUVECS in response to TNF-*α* within 15–30 min of stimulation [17]. Further, RhoA activity levels were reported to be increased after 1–5 min of TNF-*α* exposure [18, 19]. And, phosphorylation of RhoA is known to lead to the activation of ROCK, which, in turn, triggers myosin-mediated contractility via phosphorylation of the myosin light chain [19]. Hence, it is possible to test this signaling pathway by inhibiting ROCK activity.

**Fig 7:**
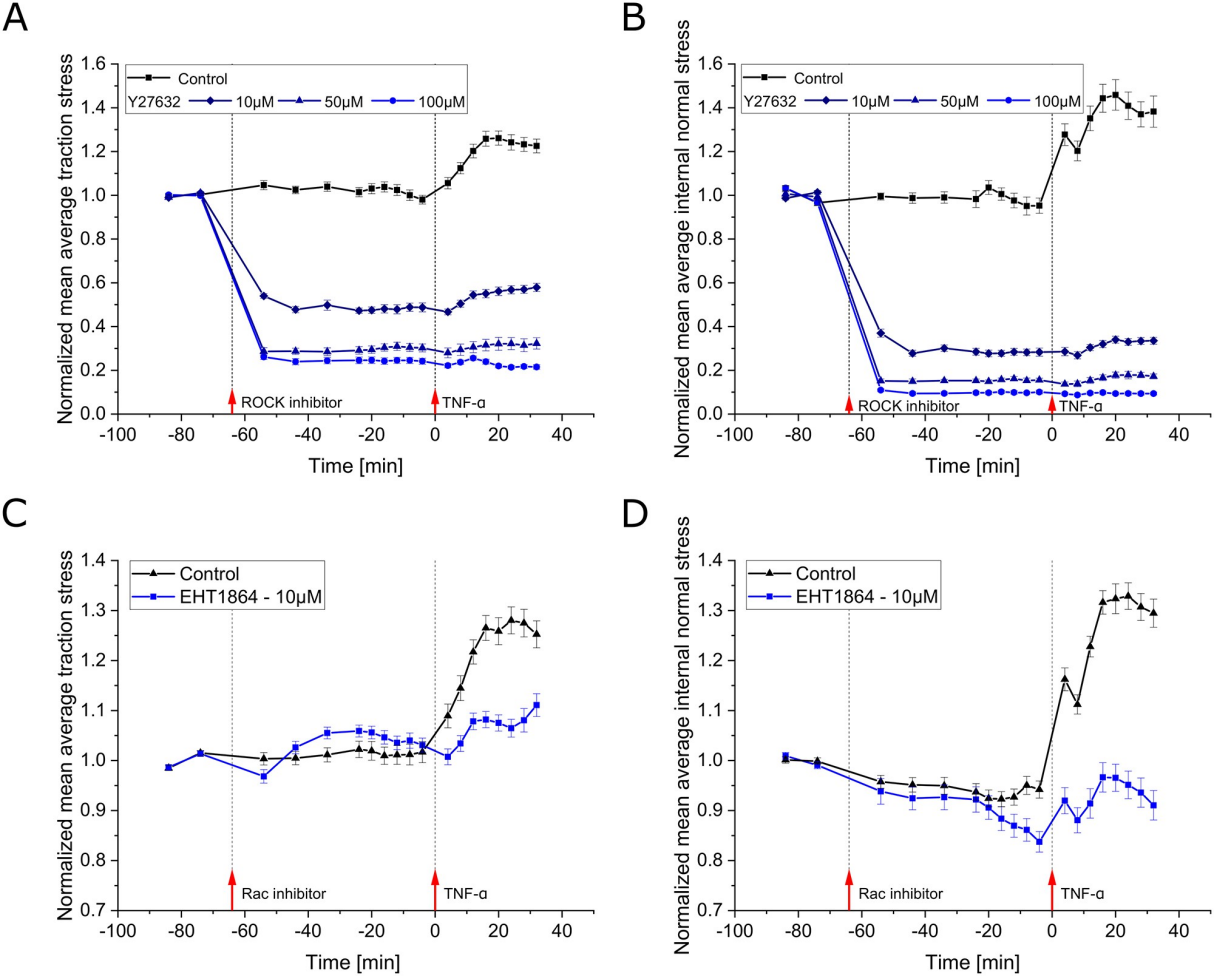
Effect of ROCK and Rac inhibitors on the mechanical response of HUVEC monolayers upon TNF-*α* stimulation. **A,B**: Time evolution of the mean of spatially averaged (A) traction stress and (B) internal normal stress of multiple monolayer islands for the control condition and 3 different concentrations of ROCK inhibitor (Y27632). Stresses were recorded at baseline, for about 1 h of ROCK inhibitor treatment and upon stimulation with 20 ng/ml TNF-*α*. Data is shown as mean ± SEM and has been normalized to the mean of the first 2 time points recorded prior to inhibitor administration for each individual monolayer island (*n*_ctrl_ = 12, *n*_10μM_ = 10, *n*_50μM_ = 7, *n*_100μM_ = 11). Inhibitor treatment causes average stresses to drop by more than 50%, reaching successively lower plateau levels with increasing concentrations. The response to TNF-*α* is progressively attenuated and rendered essentially undetectable for the highest concentration. Interestingly, the initial peak in internal normal stress observed in the control condition, which occurs due to the administration process, is nonexistent even at the lowest applied inhibitor concentration. **C,D**: Same as A and B, respectively, but for the application of the Rac1 inhibitor (EHT1864) instead of ROCK inhibitor and for only a 10 μM concentration. Three independent experiments were conducted for both control and inhibitor conditions, both recorded on the same day for the same batch of cells, respectively (*n*_ctrl_ = 33, *n*_10μM_ = 33). Average stress levels are only slightly disturbed by Rac1 inhibitor treatment. The response to TNF-*α*, however, is reduced to an amplitude similar to that observed in the ROCK inhibitor pre-treatment condition.

Consistent with this, inhibiting ROCK by increasing concentrations of the inhibitor Y27632 led to reduced average traction stress settling after about 20 min for progressively lower plateau levels (Fig 7A, for unnormalized data see S4A Fig). While control cells showed again an increase of average traction stress after TNF-*α* exposure, for inhibitor pre-treated cells this increase became successively lower with higher concentrations of ROCK inhibitor and ended up essentially undetectable. Without any obvious differences, ROCK inhibition had the same effect on the internal normal stress (Fig 7B, for unnormalized data see S4B Fig), but the initial steep rise in internal monolayer stress that was inflicted by the administration process, was fully absent. This was also the case for a 10 μM inhibitor concentration, where we still observed an overall relative increase in traction stress as well as internal normal stress after 20 min of TNF-*α* stimulation (*p* = 0.018 and *p* = 0.033, respectively, for 20 min compared to -4 min).

We next inhibited Rac1 using EHT1864, where Rac1 is known to modulate the actin cytoskeleton via the WAVE/Arp2/3 pathway [37–39]. Inhibiting Rac1 led to only a small disturbance in the average traction stress level, ending up marginally higher than in control conditions before stimulation with TNF-*α* (Fig 7C, for unnormalized data see S4C Fig). Interestingly, when TNF-*α* was added after cells were pre-treated with the Rac1 inhibitor, the rise in average traction stress observed in the control situation was suppressed to a similar degree as the suppression observed in the case of ROCK inhibition at the same concentration of the respective inhibitor. Yet, when considering the lower level of traction stress in the case of ROCK inhibition prior to TNF-*α* treatment, the relative increase following TNF-*α* stimulation was even more attenuated in the case of Rac1 inhibition. Further, the response appeared slightly delayed compared to the control situation. Regarding how Rac1 inhibition affected the internal stress response to TNF-*α*, the time evolution of the internal stress response closely matched with control condition, but the amplitude of the response was reduced to a degree similar to the observed reduction in average traction stress in the case of Rac1 inhibiton (Fig 7D, for unnormalized data see S4D Fig). Unrelated to TNF-*α* but still interesting, the initial peak in internal monolayer stress was still observed here, suggesting that a redistribution of stress, not a redistribution of the actin cytoskeleton, is responsible for the fast dynamics of the internal monolayer stress.

### Results complement former biological observations

Prior studies of early TNF-*α* stimulation by Wójciak-Stothard et al. [17] reported prominent stress fiber formation in HUVEC monolayers after 15–30 min of TNF-*α* exposure. This timing coincides with our observation of a peak in increased traction and internal monolayer stresses in response to TNF-*α* after around 20 min. Stress fiber formation has been found to follow increased MLC phosphorylation initiated by elevated levels of RhoA activity [18, 19, 40]. While RhoA activity was reported to be raised at 1–10 min of TNF-*α* exposure, enhanced levels of phosphorylated MLC were, to our knowledge, not measured before 30 min, so it remained an open question how quickly actomyosin contractility increases upon TNF-*α* treatment. Our results show a rapid increase in EC monolayer stress, that occurs immediately (within our time resolution) after stimulation, suggesting quick downstream processes following RhoA activity. Assessing TNF-*α* signaling through inhibitor application, we found that cells treated with increasing inhibitor concentrations of the ROCK inhibitor Y27632 responded successively less (and eventually did not respond) to TNF-*α* in both substrate and cell-cell forces. As ROCK is known to act downstream of RhoA, this finding underpins the role of RhoA in the mechanical response to early TNF-*α* stimulation. Besides RhoA, Rac1 has also been considered essential for early stress fiber formation in response to TNF-*α* [17]. In general, however, RhoA and Rac1 are known to have opposite effects on endothelial barrier function [41–43] and have even been found to be linked in a double-negative feedback loop in mesenchymal breast cancer cells [44]. Applying the Rac1 inhibitor EHT1864 onto our EC monolayer islands attenuated the response to TNF-*α* treatment to a similar level that the ROCK inhibitor (at the same concentration) attenuated the cell response. Yet, considering that cells treated with the ROCK inhibitor experienced a prior significant drop in overall stress levels, whereas cells treated with the Rac1 inhibitor experienced only small disturbances in stress levels, the relative increase in traction and internal monolayer stresses upon TNF-*α* stimulation was even more dampened following Rac1 inhibition. Despite the known antagonistic effects of RhoA and Rac1, these findings suggest that Rac1 is important for enhancing ECs’ contractile machinery following TNF-*α* stimulation.

## Conclusion

In the last decades, researchers have learned that endothelial cell mechanics play an important role in many inflammatory processes. In this study we uncovered the mechanical stress response of HUVECs early after TNF-*α* stimulation. In our in vitro assay, 300 μm diameter monolayer EC islands were cultured on PAA gels of 15 kPa stiffness. High traction stresses appeared at the edges of the monolayer patches and were found to be substantially transferred to neighboring cells, increasing monolayer tension toward the patches’ centers. This finding is in agreement with observations reported for other monolayer systems like epithelial cells [29]. Upon administering TNF-*α* to the cells, we found a rapid increase in average substrate traction as well as internal monolayer stress rising quickly toward a typical peak after about 20 min; this peak timing coincides with prior observations of prominent stress fiber formation [17]. The relative increase in internal normal stresses was slightly higher than that of substrate traction stresses, suggesting that TNF-*α* stimulates a particular increase in transcellular force transmission. In their natural environment, HUVECs are subjected to fluid flow, which induces mechanical shear stress. By applying a laminar medium flow at a rate causing cells to experience shear stress in the physiological range, we observed a qualitatively similar response as under the no-flow condition, but the overall relative increase in internal normal stress was significantly higher than in the no-flow condition. This might be explained by the lower base level of stresses and the higher stress cooperativity observed in the presence of flow.

While TNF-*α* is mostly known for its longer-term role in enhanced permeability and sustained leukocyte recruitment, namely at several hours post stimulation, the rapid mechanical response reported here might hint that the ECs’ contractile machinery is enhanced early on in order to support eventual leukocyte recruitment. Another possibility is that the increase in monolayer tension is simply a self-stimulating process that is needed to induce the subsequent actin cytoskeletal changes. In this case, the induced changes would then only become relevant in the long run to enable enhanced EC permeability and sustained leukocyte recruitment.

## Materials and methods

### Cell culture and treatments

Human umbilical vein endothelial cells (HUVECs) were a kind gift from Prof. Dr. V. Gerke (Institute of Medical Biochemistry, University of Münster, Germany). The cells were cultured in 60 mm diameter polystyrene dishes (Corning CellBIND Surface 60 mm Culture Dish, Corning) in culture medium consisting of two equal parts of Endothelial Cell Growth Medium 2 (PromoCell) containing its associated supplements (Growth Medium 2 SupplementMix, PromoCell) and of Medium 199 Earle’s (F0615, Biochrom GmbH or M2154, Sigma-Aldrich) with 2.2 g/l NaHCO_3_, without L-glutamine supplemented with 10% fetal bovine serum (F7524, Sigma-Aldrich), 0.2 units/ml heparin (H3149, Sigma), 30 μg/ml gentamicin (gibco) and 15 μg/ml amphotericin B (gibco) as previously described [45]. Culture dishes were placed in a humidified incubator at 37°C and 5% CO_2_. Cells were used for experiments at passages 3 to 6.

For live staining, cells were treated with CellMask Deep Red plasma membrane stain (Thermo Fisher Scientific, 1:2000). For immunostaining cells were quickly washed once with PBS^+/+^ (D8662–500ML, Sigma-Aldrich) and then fixed at indicated times in 4% paraformaldehyde at room temperature and washed after 15 min three times with PBS. Subsequently, actin filaments and nuclei were labeled using Phalloidin-iFluor 488 Reagent (Abcam, 1:1000) and Hoechst 33342 (Thermo Fisher Scientific, 1:1000), respectively, incubating the samples for 1 h in 0.2% Triton-X-100 (Carl Roth). Samples were washed again three times with PBS afterwards.

Inhibitors and the cytokine were applied at indicated concentrations and times. ROCK 1&2 inhibition was performed via Y-27632 -dihydrochlorid (Sigma-Aldrich) and Rac1 inhibition using EHT1864 (MedChemExpress). Recombinant human tumor necrosis factor-*α* was purchased from Gibco.

### PAA gel preparation with patterned coating

Polyacrylamide gels of 15 kPa stiffness (confirmed by rheological measurements and nanoindentation) were prepared as follows. To ensure stable attachment of gels to the surface of either a glass bottom dish (CELLVIEW—35mm, Greiner Bio-one International) or a glass coverslip for sticky-Slides (Ibidi), the latter were first cleaned with 0.1 N NaOH, treated with (3-Aminopropyl) trimethoxysilane (APTMS) for 3 min, thoroughly washed and then incubated with 0.5% glutaraldehyde solution for 30 min.

To create patterned surface coating of gels, 12 mm diameter round glass coverslips were first cleaned with a plasma cleaner (UV Ozone Cleaner, BioForce Nanosciences) and then incubated with 0.1 mg/ml poly(L-lysine) poly(ethylene glycol) co-polymers (PLL-PEG, SuSoS) in 10 mM HEPES solution for 1 h and washed afterwards with double-distilled water. With the help of an anti-reflective chrome-coated Quartz-glass photomask (Delta Mask B.V.), circular holes of 300 μm diameter were burned into the PLL-PEG layer using UV radiation. The circular holes were distributed over a periodic grid with 150 μm spacing (edge to edge). After quick washing, the treated sides of the coverslips were then covered for 1 h with 90 μg/ml collagen type I (Corning Collagen I, Rat Tail, Corning Life Sciences).

A gel premix was prepared by first mixing 500 μl of 40% acrylamide solution with 250 μl of 2% N,N′-Methylenebisacrylamide solution. Out of that solution, 113 μl was mixed with 372 μl of 0.65X PBS and 15 μl of fluorescent bead solution (100 nm diameter, NH_2_ coated micromer-redF, Micromod). Polymerization of gel premixes was started via 10% ammonium persulfate solution (APS) and 1.5 μl of N,N,N′,N′-Tetramethylethylenediamine (TEMED). Following quickly, 5 μl of polymerizing premix solution was put onto the surface of the glass bottom dish and covered with the pre-patterned round coverslips.

For flow experiments, polymerizing gel solution was placed on a glass coverslip with dimensions to later fit on a bottomless channel slide (sticky-Slide I Luer, Ibidi). Two tesafilm stripes were temporarily attached to the glass coverslip beforehand, and pre-patterned top coverslips were put on top of them to create a channel with a defined height and width to keep the drop of the gel solution formed such that later it properly fits into the channel of the resulting flow chamber after sticking the glass coverslip to the bottom of the sticky-Slide.

All chemicals used were purchased from Sigma-Aldrich (Merck) if not stated otherwise.

### Experimental procedure and microscopy

For all experiments, cells were seeded out 18–24 h before measurement. For experiments without flow and confluent monolayers of cells, 200 μl of a solution containing 1.6 × 10^6^ cells/ml was seeded out into 1 ml EC medium in a 35 mm diameter glass bottom dish. For single cell experiments, 3 × 10^4^ cells were seeded into the same kind of dish. After 1 h samples were washed once to remove dead cells and refilled with 2–3 ml medium. For flow experiments the channel of the flow chamber slides was filled with 150 μl of EC medium containing 1.6 × 10^6^ cells/ml. Low levels of flow were applied after 2–3 h resulting in about 0.15 Pa (1.5 dyn/cm^2^) and 0.5 Pa (5 dyn/cm^2^) shear stress for 1 h each. After that, cells were exposed to high shear stress of about 1.5 Pa (15 dyn/cm^2^) until the start of the measurement. Samples were kept in a cell culture incubator at 37°C and 5%CO_2_ until measurement.

For microscopy, samples were mounted inside a small stage top incubator (UNO Top Stage Incubator, H301-Mini, OKOLAB) on an inverted microscope (Nikon Eclipse Ti-E) and kept there again at 37°C and 5% CO_2_. The microscope was equipped with a spinning disk head (CSU-W1 Yokogawa). Images were acquired via a scientific CMOS camera (Prime BSI, Photometrics) using the Slidebook 6 software (Intelligent Imaging Innovations). Using a 40X water-immersion objective (CFI Apo LWD Lambda S 40XC WI, Nikon) with a numerical aperture of 1.15, time-lapse measurements were performed recording 3D stacks of fluorescent beads inside the gels with an excitation laser of 561 nm wavelength and of ECs using bright field illumination. For experiments on plasma membrane stained cells, a 60X water-immersion objective (CFI Plan Apo VC 60X WI, Nikon) with a numerical aperture of 1.2 and an excitation laser of 647 nm wavelength for EC images was used instead. In this case, to record the full monolayers, four 3D image stacks at four different positions with about 25% overlap were recorded for each patch and stitched together using a custom ImageJ-macro (based on [46]). Z-planes were recorded in all cases with 6 μm range à 0.33 μm interval centered around the plane most close to the gel’s surface that contained fluorescent beads. Compounds (e.g.,TNF-*α*) were applied by aspirating 2/3 of the medium from the sample dishes, mixing it with the compound and readding it. In the case of flow experiments, compounds were added to the syringe reservoirs after pausing the flow, mixed with the medium with a big pipette and applied by restarting the flow.

At the end of each time-lapse acquisition, 0.5 ml of 5% sodium dodecyl sulfate (SDS) solution was added to disintegrate the cells, and another time point was recorded as reference for no deformation.

### Traction force and monolayer stress derivation

Calculations were performed as previously reported [47]. Traction forces were derived from PAA gel deformations based on displacements of fluorescent beads close to the gel’s surface with respect to a reference state without any cell-induced deformations. The reference state was created by lysing the cells via an SDS solution. Displacements were determined by an iterative free-form deformation algorithm carried out with the open source software Elastix [48]. In short, the 3D reference image was iteratively deformed based on cubic B-spline functions and compared at each iteration to the 3D image of a respective timepoint that contained potential gel deformations. At the start of the iteration process, the control points of the B-spline functions were distributed over a regular mesh. The positions of the control points and, hence, the applied deformation was tuned in each iteration in a way that would optimize the quality of agreement between both images measured by the advanced Mattes mutual information metric. In doing so, the tuning steps followed an adaptive stochastic gradient descent algorithm. The calculation progressed in a three-level pyramid approach from the coarsest to the finest scale dividing the grid size in half and doubling the number of iterations for each level with 1.3 × 1.3 μm grid size and 2000 iterations at the finest scale. The comparison between images in each iteration was not carried out on the full images but on a randomly sampled subset of each image. The size of the subset was chosen with respect to the size of the image at hand. The final result for the deformation field was then inserted into the equation for the Tikhonov regularized inverted elasticity problem for finite thickness substrates. The equation was solved in the Fourier domain [49, 50] using a custom-made MATLAB (MathWorks) program yielding the 3D traction forces exerted onto the gel’s surface. A regularization of the equation is needed due to its inverse nature, which leads to the enhancement of high-frequency noise. Following the idea of Huang et al. [51], the Tikhonov regularization was performed combining Bayesian theory with an estimation of the background variance to provide a less subjective and more stable choice of the regularization parameter, as compared to other methods like the *L*-curve criterion or even a manual selection.

For further evaluations, the area covered by the cells was determined. In the case of stained EC monolayers, the region was inferred from the fluorescence signal via thresholding. For unstained EC monolayers, active contours [52] were applied to a manually created outline based on the 2D in-plane traction force field. The validity of the resulting monolayer outline was checked with the help of the recorded bright-field images of the cells. For single cells, the aforementioned approach led to significant discrepancies due to the discontinuity of the force distribution at the cells’ edges. Therefore, the covered region was manually created based on the bright-field images and slightly locally extended to include force spots that did not fully lie within the cell’s area due to imperfections of the force derivation. To do so, bright-field images were reversely deformed according to the determined deformation fields to match the reference frame of the calculated traction fields and overlaid with an image of the latter.

Evaluation of average traction stress values was restricted to the cell or monolayer region.

This region—created as described—was further used in the case of monolayers for the derivation of the internal stress field. Extruding this region into 3D with a 5 μm height, an object was created for finite element analysis modelling the EC monolayer as a homogenous, linear elastic material. Imposing the previously derived traction forces (only considering 2D in-plane forces) as boundary load to the bottom plane of this object, the internal stress tensor was inferred via force balance using finite element calculus performed with the COMSOL Multiphysics software package as previously described [47]. Settings used: boundary condition ‘rigid motion suppression’, material property ‘nearly incompressible’, automatically created tetrahedral mesh with ‘finer’ size. To deal with small rotational artifacts arising locally around a position that depended on the initial orientation of the object, the analysis was performed for four different orientations of the object: in-plane rotated by 90° each. For each rotation, the region exhibiting clear artifacts was omitted, and the final result obtained was an average of the results for all rotations. The internal stress results presented in this work focus on normal stresses rather than shear stresses and are given as an average of the xx- and yy-component of the derived stress tensor field.

The other components of the stress tensor, however, were used in order to calculate stress cooperativity as previously done in [27]. To quantify the stress cooperativity, the spatial autocorrelation of maximal principle stress orientations was derived as follows:

$$C(r)=\frac{1}{N}\sum_{i=1}^{N}\frac{1}{n_{i}}\sum_{j,\left|{\vec{r}}_{i}-{\vec{r}}_{j}\right|\approx r}^{N}\frac{{\vec{\sigma }}_{max,i}\cdot {\vec{\sigma }}_{max,j}}{\left|{\vec{\sigma }}_{max,i}\right|\left|{\vec{\sigma }}_{max,j}\right|}=\frac{1}{N}\sum_{i=1}^{N}\frac{1}{n_{i}}\sum_{j,\left|{\vec{r}}_{i}-{\vec{r}}_{j}\right|\approx r}^{N}\text{cos}\left(\theta_{ij}\right).$$ (1)

Here ${\vec{\sigma }}_{max,i}$, ${\vec{\sigma }}_{max,j}$ denote the maximal principle stresses considered as vector quantities at the locations ${\vec{r}}_{i}$, ${\vec{r}}_{j}$. Their orientation is defined by the angle between the axis of maximal principle stress relative to a laboratory coordinate system axis. The angle *θ*_*ij*_ then represents the angle between the two respective stress vectors. For each *i* the respective number of terms of the inner sum that fulfill the relation $|{\vec{r}}_{i}-{\vec{r}}_{j}|\approx r$ is denoted by *n*_*i*_. For the distance *r* a bin width of 5 μm was used. The outer sum runs over all *N* pixels within the monolayer region. Correlation curves were calculated for all individual monolayer islands and time points for 0–150 μm distance with a step size of 5 μm.

We defined the correlation length as the distance at a value of one half. The value was extracted from fitting the data with the function *a* * exp(−*r*/*b*) + *c* on the distance interval between 5 μm and 125 μm. The interval was restricted towards higher values to avoid regions of increasing correlation that could occur due to the symmetry of our geometry.

### Flow setup

For flow application, the pump system from Ibidi (Ibidi Pump System) was used. It consists of an electronically fine-tuned air pressure pump and a fluidic unit that holds two syringes and houses a valve that regulates the flow through the tubing. The air is taken in from a 5% CO_2_ incubator and pumped to the syringes which are connected over intertwined tubing to a flow chamber slide in a way that yields unidirectional flow through the latter while transferring fluid from one syringe to the other back and forth switching between valve states. As mentioned above, bottomless chamber slides (sticky-Slide I Luer, Ibidi) were stuck on a glass cover slip to create a flow chamber slide with 0.65 mm height, 5 mm width and 50 mm length. The PAA gels residing on the glass coverslip within the chamber had varying heights of 54–72 μm. The shear stress *τ* acting on cells at the gels’ surface was estimated via the following equation [53]:

$$\tau (x,y)=\eta \Phi /\left(\frac{4}{3}hb^{3}-8b^{4}{\left(\frac{2}{\pi }\right)}^{5}\sum_{n=0}^{\infty }\frac{1}{{\left(2n+1\right)}^{5}}\text{tanh}\left[\frac{(2n+1)\pi h}{2b}\right]\right)\times \sum_{n=0}^{\infty }\frac{{\left(-1\right)}^{n}b\pi }{{\left(2n+1\right)}^{2}}{\left(\frac{2}{\pi }\right)}^{3}\frac{\text{sinh}\left[(2n+1)\frac{\pi y}{2b}\right]}{\text{cosh}\left[(2n+1)\frac{\pi h}{2b}\right]}\text{cos}\left[(2n+1)\frac{\pi x}{2b}\right].$$ (2)

Here *η* is the viscosity of the medium, Φ the total flow through the channel, 2*h* the height and 2*b* the width of the channel. The coordinates *x* and *y* give the position centered with respect to the channel, meaning *x*, *y* = 0 at the center, *y* = −*h* at the bottom of the channel. The gel inside the chamber reduces the effective channel height to 578–596 μm. Although our gels are only 4.5 mm instead of 5 mm in width, the difference in local flow due to this variance in cross sectional area is less than 1.2%. Assuming a viscosity of *η* = 0.72 mPa ⋅ s for 37°C warm medium, the shear stress experienced by the cells at the surface of the gel lies for the highest applied flow rate of Φ = 35.66 ml/min between 1.46 and 1.64 Pa (14.6–16.4 dyn/cm^2^) dependent on the actual gel height and the position in *x*.

### Statistical analyses

Statistical comparison between two groups of data was performed either via a two-tailed *t*-test or a Mann–Whitney–Wilcoxon test depending on whether the data would qualify as normally distributed at a 5% confidence level according to a Shapiro-Wilk test. In case either or both of the two compared data sets failed to be significantly drawn from a normal distribution, the Mann–Whitney–Wilcoxon test was employed to evaluate the significance of potential differences between the compared data sets. The latter was the case for all comparisons, performed with the exception of the data shown in Fig 2B, the initial rise at 4 min in traction forces (Fig 3A), the increase at 20 min with respect to -4 min in the case of ROCK inhibitor-treated cells after addition of TNF-*α* and the data shown in S2 Fig. The Number of analyzed individual monolayer patches and number of independent repeats performed is stated in each figure caption. To test a potential difference between several groups we employed the Kruskal–Wallis test. This was done for the average correlation length for the data presented in Fig 5F where data was not normally distributed for several individual time points according to a Shapiro-Wilk test.

## Supporting information

S1 File Assessment of potential mechanical interactions between neighboring cellular islands. (PDF)

S2 File Underlying data of individual monolayer islands for all plots. (ZIP)

S1 Video Membrane stained cells display contraction upon TNF-*α* exposure. Image sequence of a representative plasma membrane stained HUVEC monolayer island about 1 h before and 1 h after the addition of TNF-*α*.
(GIF)

S1 Fig Immunofluorescence image sequence for TNF-*α* treatment. Maximum projection of representative immunofluorescence images marking the actin cytoskeleton via phalloidin and nuclei via Hoechst. TNF-*α* exposure started at *t* = 0. Scale bar, 100 μm.
(TIF)

S2 Fig Ratio of edge and inner cells’ average traction stress for flow vs. no-flow condition at peak time. Comparison of edge and inner cells’ average traction stress ratios for flow (1.5 Pa shear stress) and no-flow (zero shear stress) experimental conditions evaluated at the peak increase at 20 min past TNF-*α* addition reveals a significant difference. *n*_no_flow_ = 29, *n*_flow_ = 35, *N*_both_ = 3. **, *p* < 0.01 (*p* = 0.0016).
(PDF)

S3 Fig Unnormalized data corresponding to the experiments shown in Fig 6. (PDF)

S4 Fig Unnormalized data corresponding to the experiments shown in Fig 7. (PDF)

S5 Fig Shear instead of normal stress data corresponding to the experiment shown in Fig 3B. (PDF)
