# Supplementary material for: Human endothelial cells display a rapid tensional stress increase in response to tumor necrosis factor-α
Source: PLoS One. 2022 Jun 24;17(6):e0270197. doi: 10.1371/journal.pone.0270197 (PMC9232152; doi:10.1371/journal.pone.0270197)
Supplement: S1 File — (PDF) [file pone.0270197.s001.pdf]

Supporting information S1 File. Assessment of potential mechanical interactions between neighboring cellular islands.

To assess the potential mechanical cross-talk between neighboring cellular islands we first estimated the range to which substrate deformations caused by the cells extend beyond the region of individual monolayer islands. We calculated radial line projections of the deformation vector field and averaged over all angles (360° with 1° intervals) for each given radial distance. The adhesive pattern ends at approximately 150 µm away from the center of the individual cellular islands. As shown in Fig. A, the substrate deformation decays away from the edge of the pattern with the physically expected power law of $1/r$ in the region accessed with the microscopy images. Extending this functional dependence beyond that region (Fig. B), a deformation of zero is reached at a distance of 270 µm which is 120 µm away from the edge. As neighboring cellular islands are 150 µm apart from edge to edge, an influence between them caused by substrate deformations can hence be estimated to be negligible.

To further support this and consider other potential sources of influence, we searched for hints of periodicity in our data as the cellular islands are distributed over a periodic grid. Each pattern is surrounded by 4 direct neighbors (see Fig. C). A potential systematic influence between neighbors would therefore be expected to occur periodically every 90° around the edge of the pattern. To investigate this, we derived the normalized angular distribution of the radial projections of the deformation field of the outer 10 µm of each cellular island. We then calculated the cyclic angular auto-correlation of these projections for which a peak at 90° angular distance would be expected if measurable interaction occurs. As shown in Fig. D the resulting correlation curve does not reveal a particular increase in correlation at an angular distance of 90°.

We therefore conclude that there are no measurable interactions between neighboring cellular islands within the accuracy of our data.

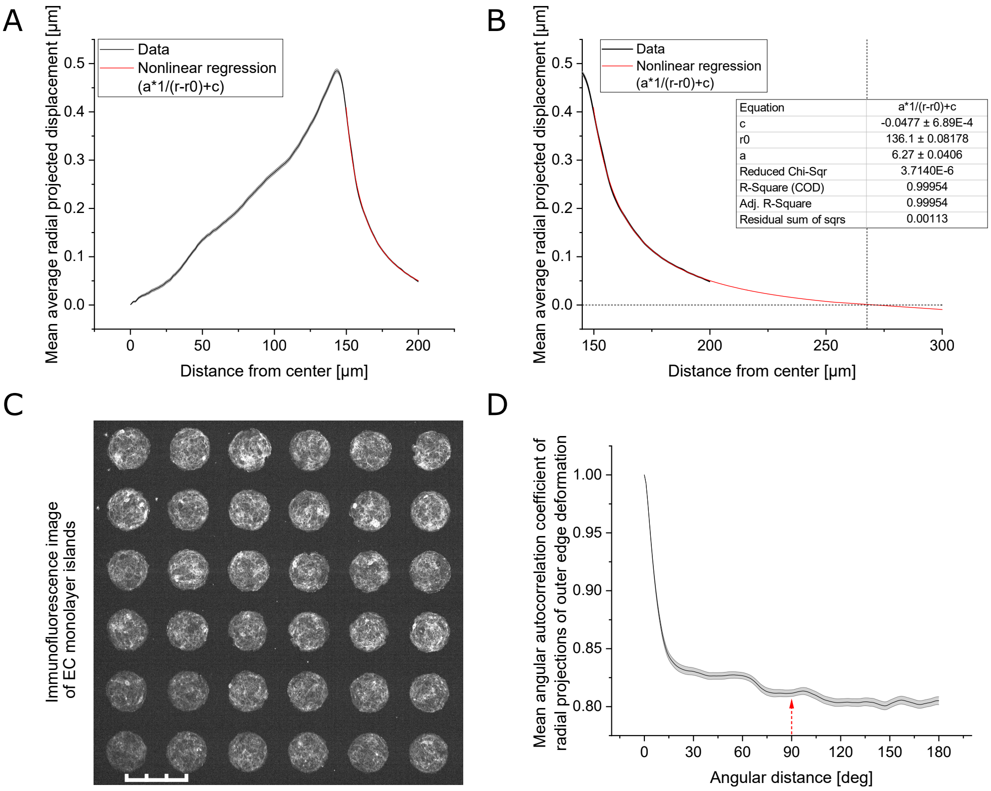

S1 Fig : **Assessment of potential mechanical interactions between neighboring cellular islands. A**: Mean profile of radially projected deformations that were averaged over all 1° angular intervals on a full circle. The mean was calculated over all individual monolayer islands from the experiment shown in Fig. 3A,B of the manuscript ($n = 29$, $N = 3$). The red line shows a nonlinear regression of the data proportional to the inverse of the radial distance r from the islands' center. **B**: Highlight of the fitted region of the plot shown in A extended to higher distances. Model parameters of the nonlinear regression are displayed in the graph. The dotted lines mark the distance where the fit crosses zero on the Y-axis. **C**: Immunofluorescene image of EC monolayer islands distributed over a regular grid with 300 µm island diameter and 150 µm edge to edge spacing. The cells' actin filaments have been labeled with a phalloidin stain. Scale bar, 450 µm with 150 µm spaced ticks. **D**: Mean angular auto-correlation coefficient of the radially projected deformations of the outer rim (projections averaged radially over the outer 10 µm) of all individual monolayer islands from the experiment shown in Fig. 3A,B ($n = 29$, $N = 3$). The auto-correlation coefficients have been calculated in a circular manner, i.e. angular distances were calculated modulus 360°. The underlying angular profiles of the radial projections of the deformations have been calculated with 1° intervals. The radial projections were normalized by their angular average for each individual monolayer island, respectively. The gray band shows the standard error of the mean. The red arrow marks 90° angular distance at which an increase of the correlation would be expected in case of influence between neighboring cell patterns on a periodic rectangular grid.
