# Supplementary figures and images for: Human endothelial cells display a rapid tensional stress increase in response to tumor necrosis factor-α
Source: PLoS One. 2022 Jun 24;17(6):e0270197. doi: 10.1371/journal.pone.0270197 (PMC9232152; doi:10.1371/journal.pone.0270197)

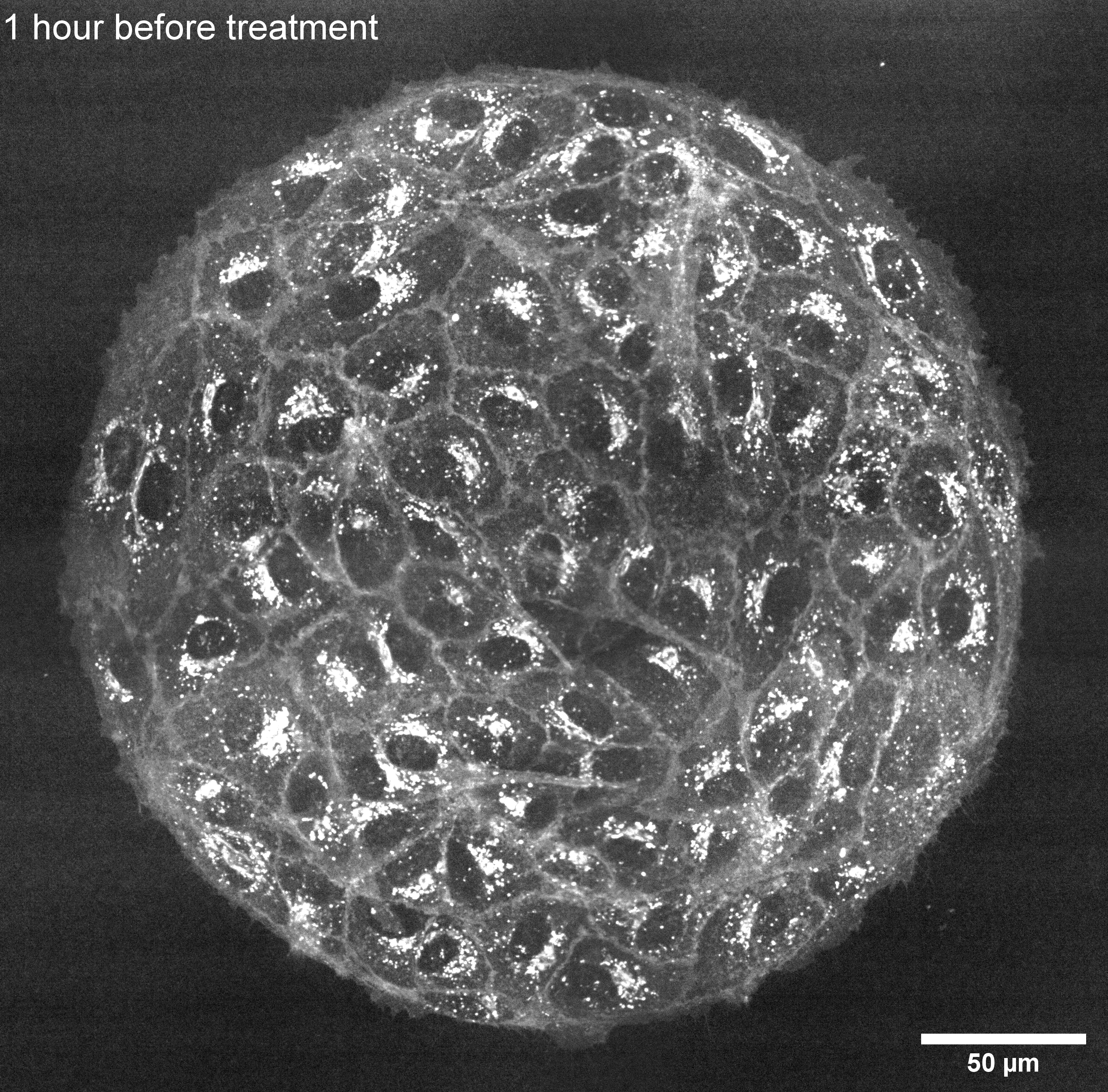

Supplement: S1 Video — Image sequence of a representative plasma membrane stained HUVEC monolayer island about 1 h before and 1 h after the addition of TNF-α. (GIF) [file pone.0270197.s002.gif]

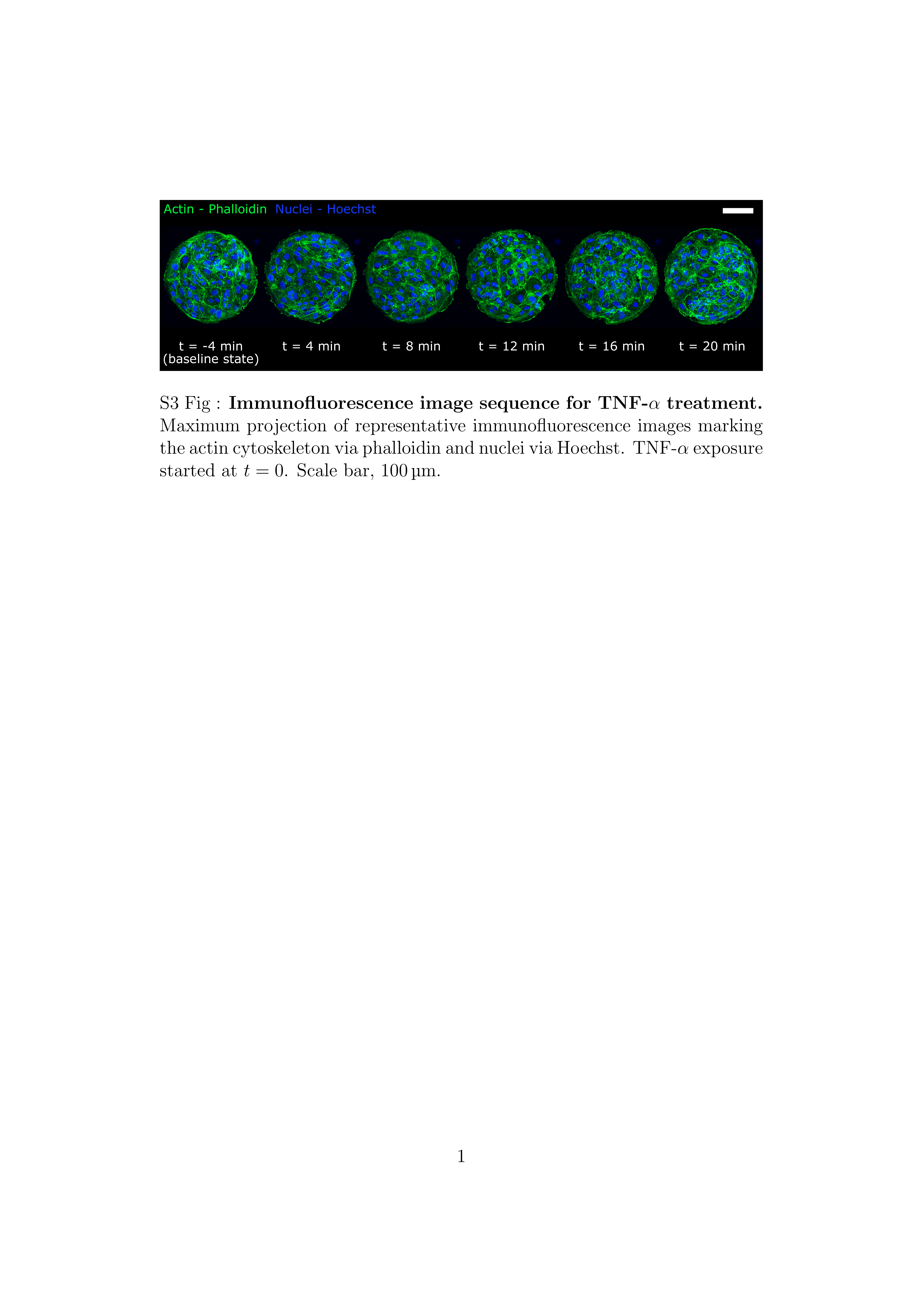

Supplement: S1 Fig — Maximum projection of representative immunofluorescence images marking the actin cytoskeleton via phalloidin and nuclei via Hoechst. TNF-α exposure started at t = 0. Scale bar, 100 μm. (TIF) [file pone.0270197.s003.tif]

A

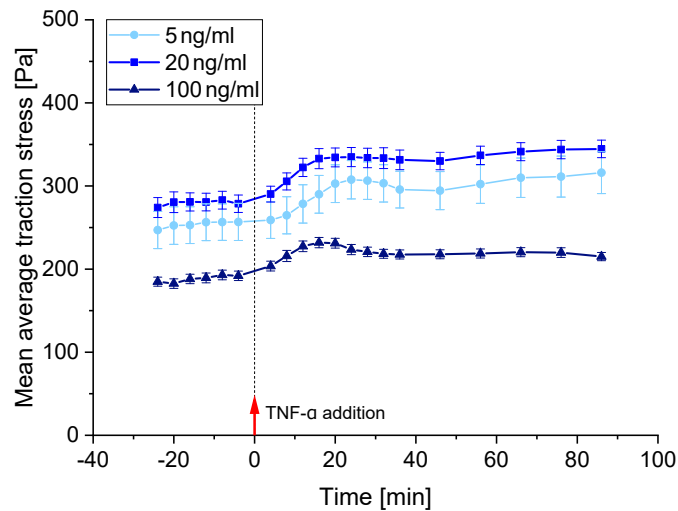

B

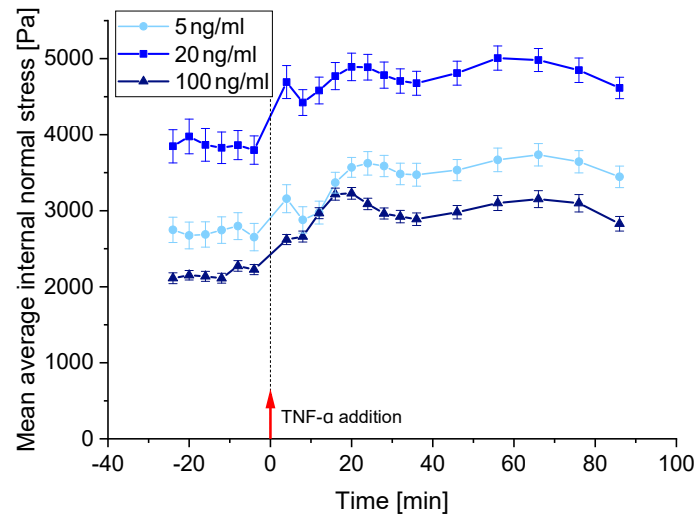

C

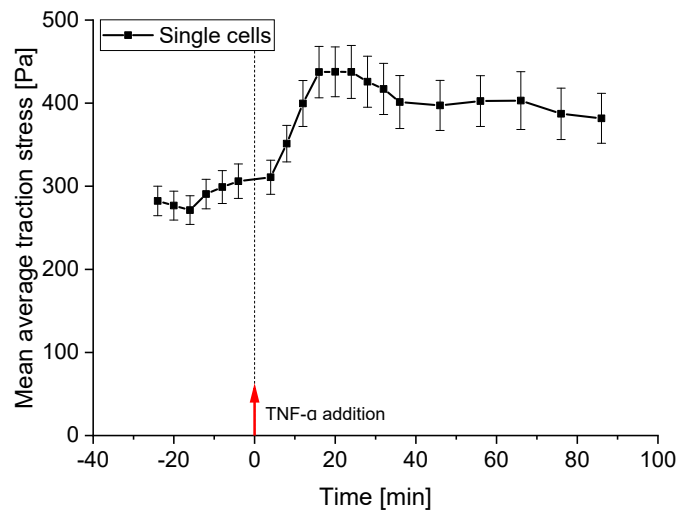

D

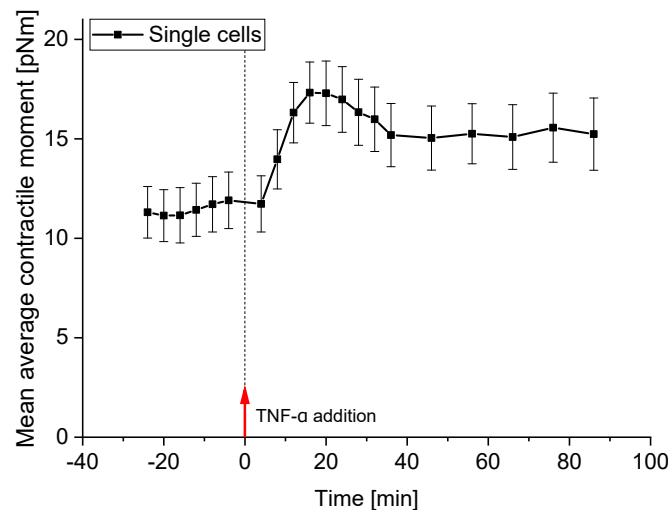

Supplement: S3 Fig — (PDF) [file pone.0270197.s005.pdf]

A

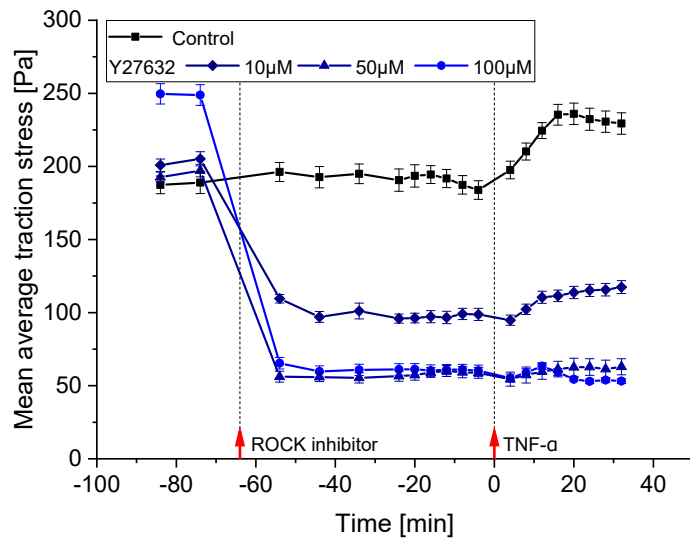

B

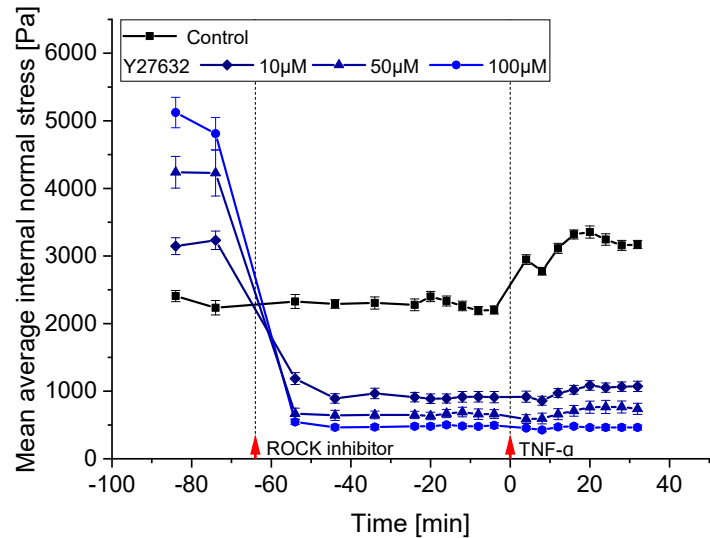

C

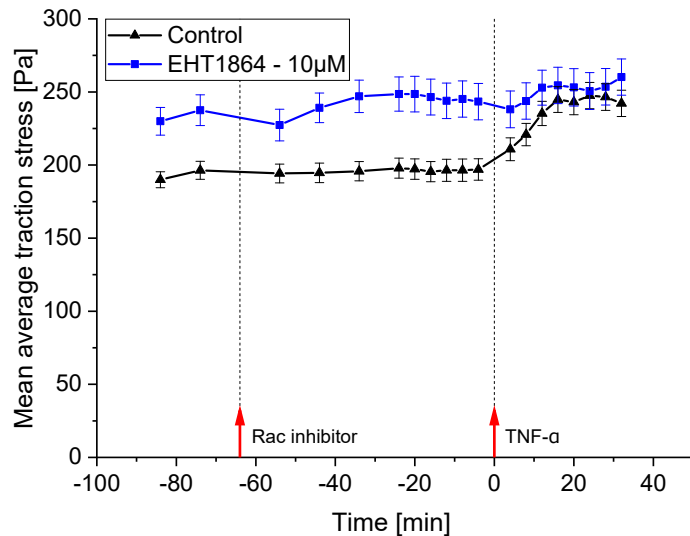

D

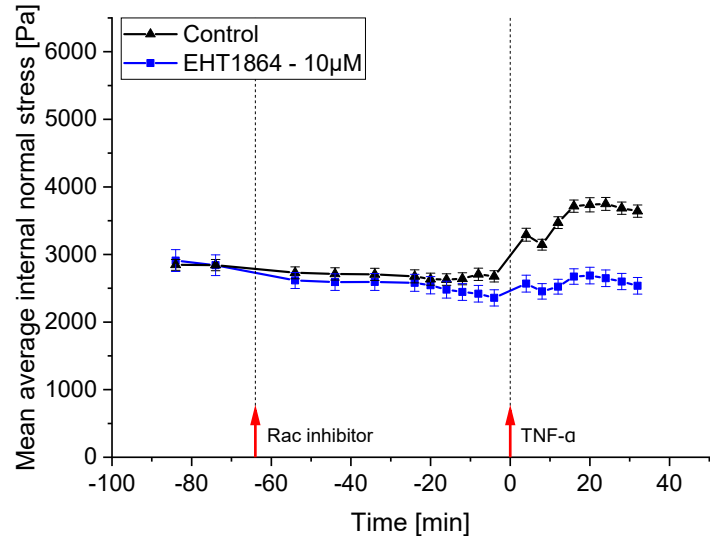

Supplement: S4 Fig — (PDF) [file pone.0270197.s006.pdf]

A

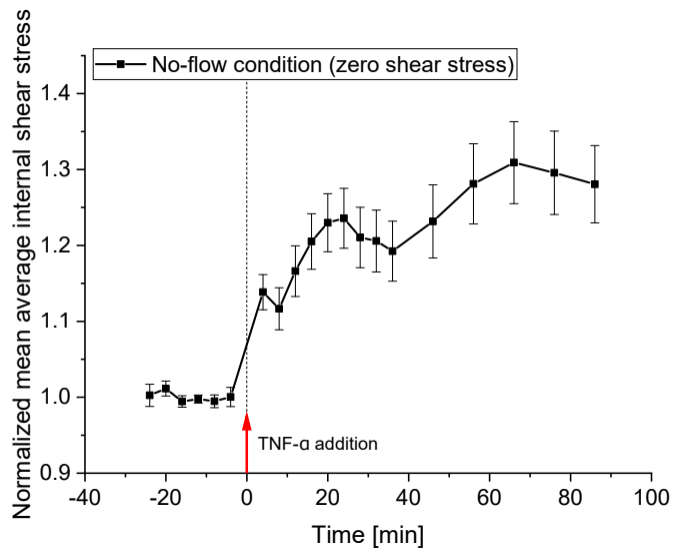

B

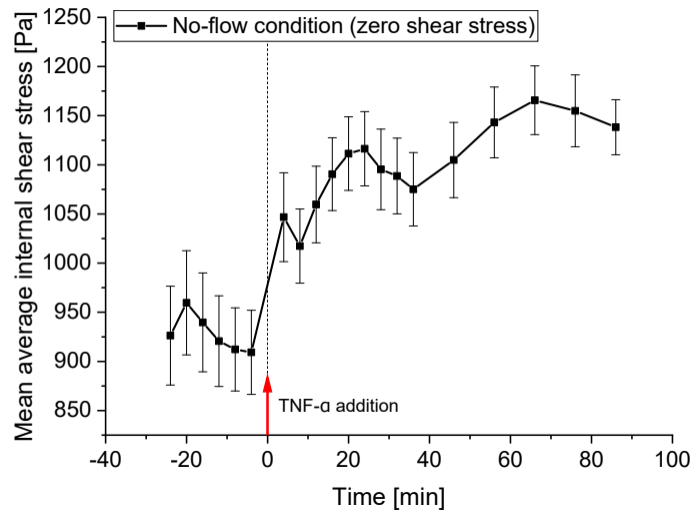

Supplement: S5 Fig — (PDF) [file pone.0270197.s007.pdf]
