# Supplementary material for: Human endothelial cells display a rapid tensional stress increase in response to tumor necrosis factor-α
Source: PLoS One. 2022 Jun 24;17(6):e0270197. doi: 10.1371/journal.pone.0270197 (PMC9232152; doi:10.1371/journal.pone.0270197)
Supplement: S2 Fig — Comparison of edge and inner cells’ average traction stress ratios for flow (1.5 Pa shear stress) and no-flow (zero shear stress) experimental conditions evaluated at the peak increase at 20 min past TNF-α addition reveals a significant difference. nno_flow = 29, nflow = 35, Nboth = 3. **, p < 0.01 (p = 0.0016). (PDF) [file pone.0270197.s004.pdf]

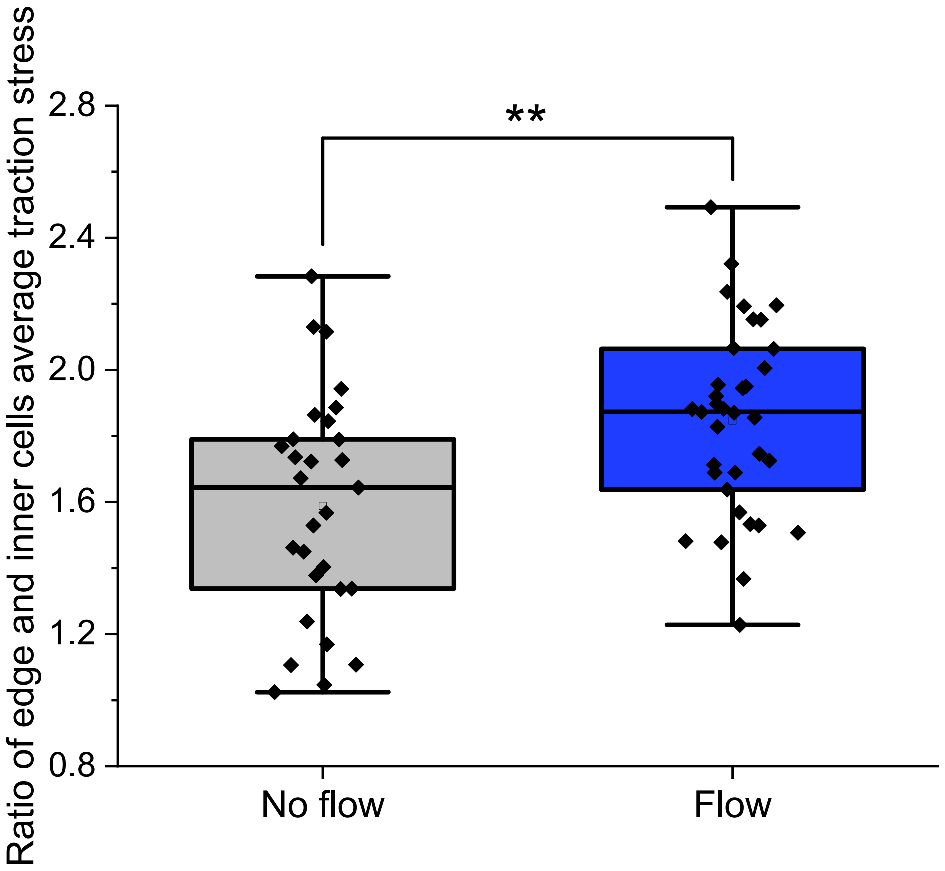

S4 Fig : **Ratio of edge and inner cells' average traction stress for flow vs. no flow condition at peak time.** Comparison of edge and inner cells average traction stress ratios for flow (1.5 Pa shear stress) and no-flow (zero shear stress) experimental conditions evaluated at the peak increase at 20 min past TNF-α addition reveals significant difference. $n_{\text{no\_flow}} = 29, n_{\text{flow}} = 35, N_{\text{both}} = 3$. **, $p < 0.01$ ($p = 0.0016$)..
